# It's a hard knock life for some: Heterogeneity in infection life history of salmonids influences parasite disease outcomes

**DOI:** 10.1111/1365-2656.13562

**Published:** 2021-07-21

**Authors:** Christyn Bailey, Nicole Strepparava, Albert Ros, Thomas Wahli, Heike Schmidt‐Posthaus, Helmut Segner, Carolina Tafalla

**Affiliations:** ^1^ Fish Immunology and Pathology Group Animal Health Research Centre (CISA‐INIA) Madrid Spain; ^2^ Centre for Fish and Wildlife Health University of Bern Bern Switzerland; ^3^ LAZBW Fischereiforschungsstelle Langenargen Germany

**Keywords:** disease ecology, host heterogeneity, host–parasite interaction, immunity, infection life history, proliferative kidney disease

## Abstract

1. Heterogeneity in immunity occurs across numerous disease systems with individuals from the same population having diverse disease outcomes. Proliferative kidney disease (PKD) caused by *Tetracapsuloides bryosalmonae*, is a persistent parasitic disease negatively impacting both wild and farmed salmonids. Little is known of how PKD is spread or maintained within wild susceptible populations.
2. We investigated an aspect of fish disease that has been largely overlooked, that is, the role of the host phenotypic heterogeneity in disease outcome. We examined how host susceptibility to *T. bryosalmonae* infection, and the disease PKD, varied across different infection life‐history stages and how it differs between naïve, re‐infected and persistently infected hosts.
3. We investigated the response to parasite exposure in host phenotypes with (a) different ages and (b) heterogeneous infection life histories. Among (a) the age phenotypes were young‐of‐the‐year (YOY) fish and juvenile 1+ fish (fish older than one) and, for (b) juvenile 1+ infection survivors were either re‐exposed or not re‐ exposed to the parasite and response phenotypes were assigned post‐hoc dependant on infection status. In fish not re‐exposed this included fish that cleared infection (CI) or had a persistent infection (PI). In fish re‐exposed these included fish that were re‐infected (RI), or re‐exposed and uninfected (RCI). We assessed both parasite‐centric (infection prevalence, parasite burden, malacospore transmission) and host‐centric parameters (growth rates, disease severity, infection tolerance and the immune response).
4. In (a), YOY fish, parasite success and disease severity were greater and differences in the immune response occurred, demonstrating an ontogenetic decline of susceptibility in older fish. In (b), in PI and RI fish, parasite success and disease severity were comparable. However, expression of several adaptive immunity markers was greater in RI fish, indicating concomitant immunity, as re‐exposure did not intensify infection.
5. We demonstrate the relevance of heterogeneity in infection life history on disease outcome and describe several distinctive features of immune ontogeny and protective immunity in this model not previously reported. The relevance of such themes on a population level requires greater research in many aquatic disease systems to generate clearer framework for understanding the spread and maintenance of aquatic pathogens.

## Graphical abstract

Recently we have all become aware that different individuals react to disease in a different ways, resulting in diverse outcomes. This also happens underwater for aquatic wildlife in terms of how infection life‐history influences the immune response and infection rates within a population and contributes to the spread and maintenance of disease.

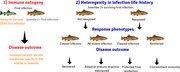

## 1 INTRODUCTION

Hosts have established a mosaic of physiological responses to manage parasite infections. In essence, a host population is divided into susceptible individuals, clinically infected individuals and protected individuals, distributed across various age classes (Hudson et al., 2008; Pfennig, 2001; Schmid‐Hempel & Koella, 1994). The outcome between a host and a parasite can be shaped by the age of the host, the infection life history and the environment. By the term infection life history, we refer to either the lack of, or the abundance of parasite encounters and infection outcomes of the host. In this setting, if a form of protective immunity has been established due to a previous or persistent infection, it will then influence the outcome of a subsequent encounter in terms of the host immune response elicited, the parasite burden, the disease pathogenesis and the host´s ability to allocate metabolic resources to growth or reproduction during infection (Penczykowski et al., 2016). In fact, heterogeneity in host immune status and infection intensity has been described in numerous disease systems showing that individuals from the same population with diverse infection life histories have different disease outcomes (Bradley et al., 2019; Coltman et al., 1999; Hart, 1994; Hudson et al., 2008; Johnson & Hoverman, 2014; Wilson et al., 2002). For instance, increased age has been reported to both increase and/or decrease infection prevalence and infection intensity in different host–parasite systems (Bundy et al., 1987; Duerr et al., 2003; Gregory, 1992; Mpofu et al., 2020; Phillips et al., 2020). Such considerations are essential to envisage how host susceptibility and immunity might be impacted by parasitism.

A growing threat to cultured and wild salmonids in North America and Europe is the parasitic disease proliferative kidney disease (PKD), caused by the myxozoan *Tetracapsuloides bryosalmonae*. PKD is one of several fish diseases sensitive to the ongoing climate crisis (Marcos‐López et al., 2010; Okamura et al., 2011). Multiple lines of evidence have indicated that both incidence and severity of PKD are exacerbated by rising temperatures (Bailey et al., 2017; Bettge et al., 2009; Bettge et al., 2009; Carraro et al., 2017; Okamura et al., 2011; Rubin et al., 2019). In Europe and North America, farmed salmonids are strongly affected by PKD in which outbreaks can reach 90% mortality (Clifton‐Hadley et al., 1986; Okamura et al., 2011). PKD also affects wild fish and is suggested to be responsible for the long‐term decline in salmonid populations in European countries, most notably the brown trout populations in Switzerland (Mo & Jørgensen, 2017; Wahli et al., 2002). Still, little is known of how PKD is spread or persists in wild populations or how the disease affects the different individuals that make up susceptible populations.

*T. bryosalmonae*, the PKD‐causing parasite has a two‐host life cycle encompassing freshwater bryozoa as invertebrate hosts and mainly salmonid fish as vertebrate hosts. In bryozoa, *T. bryosalmonae* malacospores develop and when released infect susceptible fish hosts through the gills and skin, from where they are transported via the blood to the target organs for colonization (Grabner & El‐Matbouli, 2008). In the posterior kidney, the main target organ, the parasite proliferates and produces malacospores that when released are infective to bryozoans, but not fish, for example, no horizontal transmission occurs, thus completing the life cycle (Fontes et al., 2017; Grabner & El‐Matbouli, 2008). Due to the immunologically active nature of the fish posterior kidney, parasite development provokes a chronic lymphoid immunopathology, and a massive swelling develops. Additionally, parasites can invade and cause an immune reaction in other organs, including the anterior kidney, spleen and liver (Bailey et al., 2020; Hedrick et al., 1993; Okamura et al., 2011). In natural conditions naïve fish are continuously exposed to the parasite in the summer months for the first time as young‐of‐the‐year (YOY ‐ aged 0+ fish, those animals born within the current year) and are confronted again by the parasite in the subsequent years.

Much of the knowledge of host immunity during PKD pathogenesis is generated from studies using a model species, the non‐native rainbow trout *Oncorhynchus mykiss*, exposed to the European strain of the parasite for which it acts as a dead‐end host (Abos et al., 2018; Bailey et al., 2017, 2019, 2020; Bailey, Segner, & Wahli, 2017; Chilmonczyk et al., 2002; Gorgoglione et al., 2013; Granja et al., 2017). This emphasis on rainbow trout is due to the severe economic constraints PKD places on the rainbow trout industry in Western Europe (Okamura et al., 2011). Hence, insight of rainbow trout immunity is imperative to guide the design of specific therapeutics and vaccines suitable for immunization. In this species, a huge B cell proliferation and dysregulation with aberrant immunoglobulin production and plasma cell differentiation occurs as well as an imbalance of Th‐like cytokines (Abos et al., 2018; Bailey, Holland, et al., 2020; Bailey, Segner, Casanova‐Nakayama, et al., 2017; Bailey, Segner, et al., 2020; Gorgoglione et al., 2013). In brown trout *Salmo trutta*, a species in Europe that has co‐evolved with *T. bryosalmonae* in which the parasite can fulfil its life cycle, some knowledge generated on the transcriptional level has described increased expression of some B cell transcripts and Th1‐like cytokines in parasite‐infected fish (Bailey et al., 2019; Kumar et al., 2014, 2015; Sudhagar et al., 2019). While dysregulation of B and T cell responses are shared in both species, some differences in the intensity and sequential aspects of the immune response have also been reported (Bailey, Strepparava, et al., 2019; Sudhagar et al., 2019).

Thus, while recent research has identified generalities in the immune response against *T. bryosalmonae*, we still have a limited understanding to what extent there exist heterogeneities in the PKD immune response between different host phenotypes. Furthermore, and critical in the context of wild fish, knowledge is lacking on how these differences may influence disease outcome, spread and maintenance within and between species. For example, a field study described juvenile brown trout (1+ those aged between 1 and 2 years old) having lower infection prevalence and less pathology compared to YOY brown trout (Schmidt‐Posthaus et al., 2013). Nevertheless, it was unknown if this difference in infection outcome was related to age or protective immunity. In the dead‐end host, the rainbow trout, when exposed to the European strain, this host can recover completely from infection, and establish a form of protective immunity (Bailey, Segner, et al., 2020; Schmidt‐Posthaus et al., 2012). However, in the co‐evolved system, while a long‐term persistent infection has been documented (Soliman et al., 2018), it is unknown to what extent brown trout can establish protective immunity. This is a question that must be approached from multiple angles using fish with heterogeneous infection life histories, for example, how fish react to re‐infection either with a persistent infection or in a fish that cleared the infection and is re‐exposed.

The brown trout—*T. bryosalmonae* PKD disease model presents itself as an ideal and relevant system to investigate host‐parasite dynamics because (a) we have an overview of fish host immune response during PKD pathogenesis (Bailey, Holland, et al., 2020), (b) PKD is a serious disease impacting on a conservation and economical level (Okamura et al., 2011), (c) *T. bryosalmonae* infection intensity and PKD pathogenesis are exacerbated by the ongoing climate crisis and epidemiological models have predicted both an increase in severity and incidence (Carraro et al., 2016), and (d) PKD is a chronic infection providing an opportunity to follow the host response over longer periods, which is not possible for many other fish bacteria or viral models that cause high instantaneous mortalities.

We investigated an aspect of fish disease that has been largely overlooked, that is, the role of the host phenotypic heterogeneity in disease outcome. We examined how susceptibility to *T*. *bryosalmonae* infection, and the disease PKD, varied across different infection life‐history stages. The brown trout was selected as our model species, due to the species co‐evolutionary history with the European strain of *T. bryosalmonae*. We separated our analysis into two research questions one focusing on the impact of immune ontogeny on infection and one focusing on protective immunity. To investigate immune ontogeny, we compared disease progression and outcome in fish with different age classes infected for the first time, this included YOY fish, and juvenile 1+ fish (those fish born the previous year aged between 1 and 2). To investigate protective immunity, we studied juvenile 1+ fish surviving their first infection as YOY fish. These fish were placed into experimental groups that would either be re‐exposed or not re‐exposed. We studied the host–parasite interaction for 80 days post exposure (dpe). We assessed both parasite‐centric parameters: *T. bryosalmonae* infection prevalence, parasite intensity and fish malacospore release; and host‐centric parameters: fish growth, disease severity, host tolerance and the adaptive immune response. This included measuring the three fish Igs, *igm*, *igt* and *igd*, in terms of both membrane and secreted isoforms as well as *blimp1* the B cell master regulator that drives B cell differentiation to plasma cells. We predicted (a) that the host response to parasite infection is dependent on the host's age, and that older fish will tolerate infection better and (b) that while the majority of parasite‐centric parameters and host‐centric parameters will be comparable between fish with a persistent infection and fish re‐infected, we expected differences to occur in the immune response exhibited by the different phenotypes.

## 2 MATERIALS AND METHODS

### 2.1 Fish specimens

To compare the host response in different age classes YOY brown trout (*n* = 610) weighing on average 3.4 ± 0.4 g and juvenile 1+ brown trout aged over 1 year of age (*n* = 460) weighing on average 58 ± 22 g, were obtained from a hatchery with no history of PKD in southern Switzerland (Pescicoltura di Maggia, Maggia, Switzerland). Fish at the hatchery are produced for stocking and the fish farm always maintains a source of parent fish, which these fish were bred from, thus there is a shared genetic background between YOY and juvenile 1+ fish. On arrival, 10 fish were screened for parasites (including *T. bryosalmonae* by qPCR see methods 2.6) by microscopical assessment of gill and skin scrapings and intestinal content and for bacterial agents by culture on blood agar plates as previously described (Anacker & Ordal, 1959). Additional viral screenings were performed by standard cell culture methods as previously described (Bailey et al., 2018). No infectious agents were detected.

To investigate if heterogeneity in host infection life history modulated *T. bryosalmonae* infection, juvenile 1+ brown trout PKD survivors (*n* = 450) weighing on average 47 ± 17 g were recovered from a previous experiment (Strepparava et al., 2020). These fish were obtained from the same hatchery as the naive specimens. In this previous experiment, brown trout were used to study the impact of different parasite concentrations upon infection dynamics. In which three concentrations were used, a low concentration, high concentration and repeated exposure treatment (Strepparava et al., 2020). While the low concentration treatment resulted in some reduced outcomes across infection parameters, infection prevalence reached 100% in all treatments and gross parasite burdens and mortality were similar (Strepparava et al., 2020).

### 2.2 Establishment of experimental phenotypes

#### 2.2.1 Hosts with different age classes

**FIGURE 1:**
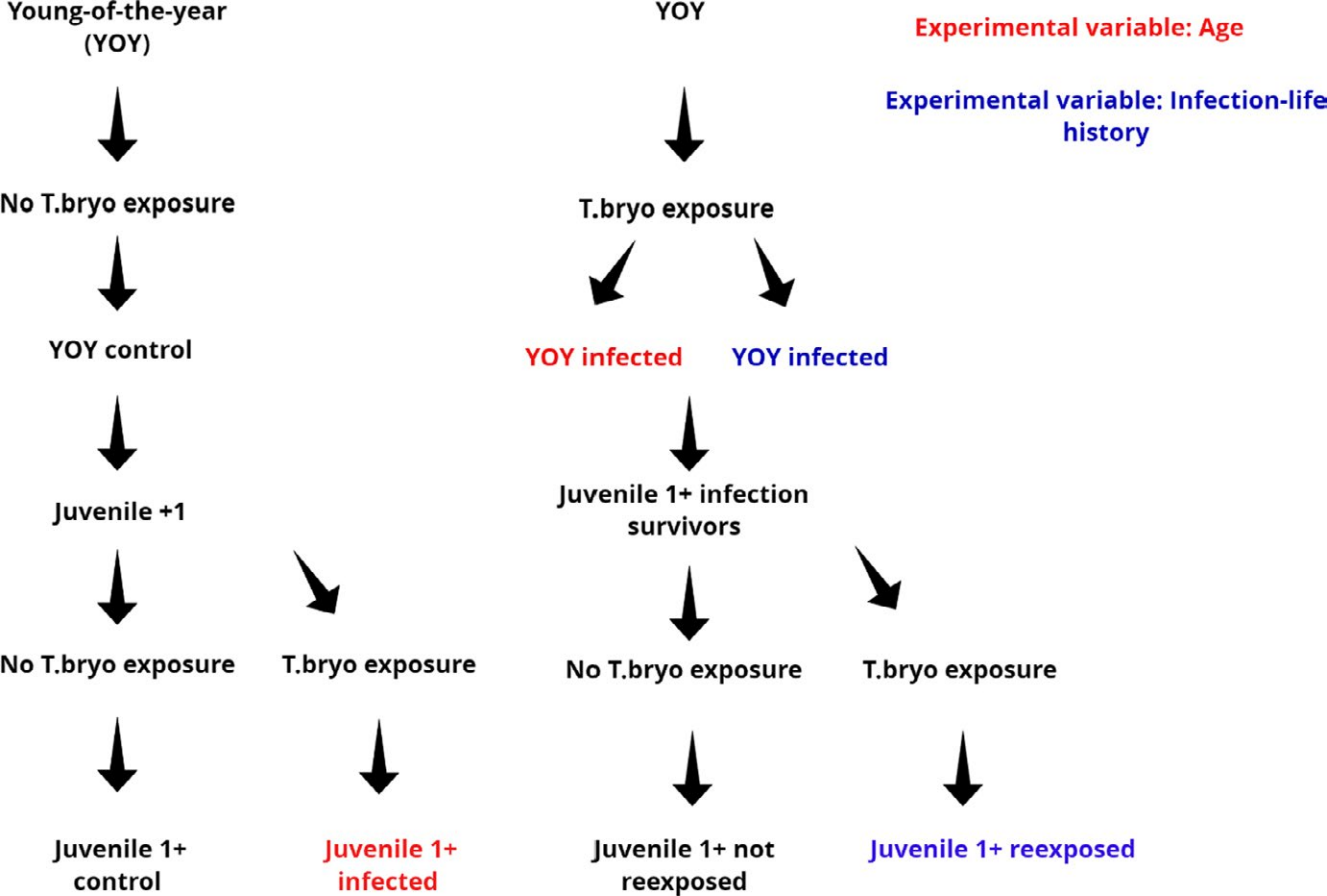
Flow diagram detailing the establishment of experimental treatments, used in both the age classes assessment (red) and in the assessment of hosts with heterogeneous infection life histories either re‐exposed or not re‐exposed to the parasite (blue). T. bryo = *Tetracapsuloides bryosalmonae*

**FIGURE 2:**
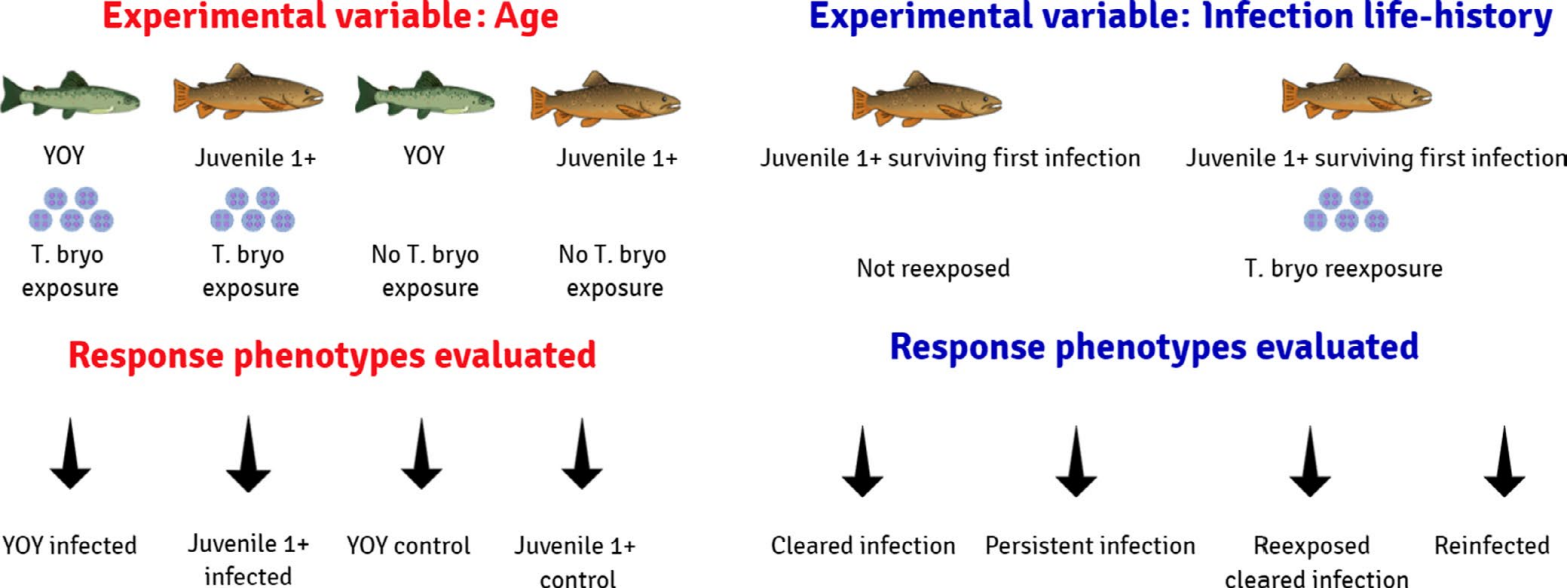
Schematic of the response phenotypes evaluated used in both the age classes assessment (red) and in the assessment of hosts with heterogeneous infection life histories (blue). YOY = young‐of‐the‐year, T. bryo = *Tetracapsuloides bryosalmonae*

The establishment of all experimental groups is represented in Figure 1, while the establishment of the response groups that were evaluated is described in Figure 2. Concerning the hosts with different age classes, both the YOY fish and the juvenile 1+ fish were separated in two groups each: YOY control and YOY infected, with two replicates tanks of each (*n* = 150 fish per replicate; Figure 1) and juvenile 1+ control and juvenile 1+ infected, but with three replicates each of (*n* = 75 fish per replicate due to their bigger size). While juvenile 1+ control is not a phenotype that would likely occur in a field scenario it is a necessary control for us to test the effect of immune ontogeny and to investigate the role of protective immunity.

#### 2.2.2 Hosts with heterogeneous infection life histories

The fish surviving an earlier *T. bryosalmonae* infection were also separated into experimental groups. These included a group that would not be re‐exposed and a group to be re‐exposed to the parasite, also with three replicates each (*n* = 75 fish per replicate; Figure 1). All these fish were juveniles older than 1 year, and their original infections occurred 1 year ago. Response groups that would be evaluated were assigned post‐hoc after assessment of infection status, via qPCR and pathological evaluation (see methods Sections 2.6 and 2.7; Figure 2). These included from those fish not re‐exposed (a) fish that had cleared infection (CI ‐ cleared infection) and (b) fish that had a persistent infection (PI ‐ persistent infection); from fish re‐exposed (c) fish that had cleared the infection (RCI ‐ re‐exposed cleared infection) and (4) fish that were re‐infected (RI ‐ re‐infected; Figure 2). All these treatments were evaluated and compared for all parameters. Juvenile 1+ control fish (see Section 2.3.2) were considered the absolute uninfected control for these experimental groups as they were of the same age and source and pathogen free.

### 2.3 Fish maintenance

All fish were maintained at 15 ± 1℃ (*M* ± *SE*) and acclimated for 2 weeks in the experimental aquaria. All experiments were run in flow through glass tanks with a volume of 130 L supplied with tap water (1 L/min) and constant aeration. Mortalities were recorded daily; fish dying between the sampling days were necropsied and investigated for presence of parasites or other infectious agents. Approval for animal experiments was obtained from the Cantonal Veterinary Office (Bern, Switzerland; Authorization BE11/16).

### 2.4 Collection of *T. bryosalmonae* populations for fish exposure

To obtain the parasites for exposures, *T. bryosalmonae*‐infected bryozoans (*Fredericella sultana* only, identified using morphology, Wood & Okamura, 2005) were obtained from four Swiss rivers as previously described (Strepparava et al., 2020). The populations of *T. bryosalmonae*‐infected bryozoans were kept in ≥8 L of river water in a 10‐L bucket at room temperature for 3 days enabling the continuous release of malacospores (Strepparava et al., 2020). After 12 hr, qPCR was carried out on 2 × 100 ml replicate samples of water from the bucket to confirm that malacospores were released from the bryozoans in the water (see Section 2.6 for detailed qPCR description). On day 3, all the bryozoans were disrupted to further allow malacospore release for exposure. Prior to infection the water flow in all tanks was stopped and the water level reduced to ≈30 L and aeration was strengthened. The 8‐L homogenate containing the released malacospores spores and disrupted bryozoan tissue were thoroughly mixed and distributed according to number of fish per exposure tanks. For example, YOY infected tanks had 150 fish per replicate tank this meant they received double the infection homogenate than that of the juvenile 1+ infected replicates which contained 75 fish per replicate tank. The identical procedure was applied to the respective controls without any addition of the infective solution. After 1.5 hr after the water flow was restarted.

To confirm that the infective dose between the tanks was similar, water samples (2 × 100 ml) were taken from each replicate tank 20 min post exposure. The water samples were filtered using a 0.25‐µm nitrocellulose filter (Merck Millipore) and papers stored at −20℃. The parasite DNA concentration in the water was estimated using DNA extraction from filter papers and real‐time quantitative PCR (qPCR). All samples collected from water tanks during the initial fish exposure displayed homogenous parasite DNA at an average (*M* ± *SE*) concentration of 4.8 × 10^5^ ± 10% copies per fish.

Past PKD studies have used unlimited and/or continuous doses of spores often resulting in excessive mortality with no control of the exact dose (Bettge, Segner, et al., 2009; Bettge, Wahli, et al., 2009), whereas in our controlled experimental model, a defined equal spore concentration is used (Strepparava et al., 2018, 2020). Our model still results in clinical PKD, but eliminates excess mortality and continuous infection, allowing us to explicitly focus on host infection processes in a controlled fashion (Bailey, von Siebenthal, et al., 2019; Strepparava et al., 2018, 2020). With a continuous exposure, all changes of infection processes in the host would be a mixture of within‐host proliferation and new infections.

### 2.5 Fish sampling process

Fish were sampled at 0, 10, 20, 30, 50 and 80 days post exposure (dpe) from all experimental groups. From each YOY tank (YOY control and YOY infected), 10 fish were sampled per replicate (*n* = 20 per experimental group), in all the other treatments (juvenile 1+ control and juvenile 1+ infected) and in the re‐exposure study, seven fish were sampled (*n* = 21 per experimental group) at each time point. From these fish, the post‐hoc qPCR and histopathology assessments indicated which fish were either CI or PI and RI or RCI response phenotypes. This resulted in a minimum of at least six fish to use in these groups per time point. Sampled fish were euthanized using 3‐aminobenzoic acid ethyl ester (MS 222^®^, Argent Chemical Laboratories). Following this, fish length (L) and weight (W) were recorded to calculate the specific growth rate (SGR (ln (final weight in grams) − ln (initial weight in grams) × 100)/t (in days)) and the posterior kidney removed and weighed to calculate the posterior kidney somatic index (PKSI). The PKSI was calculated as: posterior kidney weights (g)/body weights (g) × 100. The posterior kidney was then cut in two halves longitudinally: One half was homogenized in 1.5 ml TRI solution (Sigma) for future RNA/DNA isolation and the other half was placed in formalin to be prepared for histology procedures. All parameters were assessed at every time point apart from fish malacospore release as this did not occur till later in the experiment (see Section 2.5) and the RT‐qPCR immune gene expression analysis, which was performed at 10, 50 and 80 dpe.

### 2.6 Assessment of fish malacospore release

To evaluate when infected fish started releasing malacospores post exposure, water samples were collected from all replicate tanks during the experiment and analysed for the presence of *T. bryosalmonae* DNA as an estimate of the parasite material released from the fish indicating the release of fish malacospores (Morris & Adams, 2006). Our earlier work found that fish malacospore release began at 45 dpe (Strepparava et al., 2020), thus we began collecting water samples slightly earlier than this, at 30 dpe. This was performed to (a) corroborate our previous research and (b) to examine if fish malacospore release was continuous in the re‐exposed fish. We continued collecting water samples every 10 days from 45 dpe to 75 dpe prior to the termination of the study at 80 dpe using the same method established in our earlier studies. In short, 2 × 1 L water samples from each tank were filtered using a 3.0‐μm nitrocellulose filter (Merck Millipore) which was stored at −20℃ until further processing (Strepparava et al., 2018, 2020).

### 2.7 DNA isolation quantification of qPCR to determine parasite intensity

Genomic DNA was isolated from the brown trout posterior kidney of all specimens as previously described (Harun et al., 2011; Strepparava et al., 2020) and eluted in 30 µl of EB buffer (QIAGEN) and stored at −20℃ until processing for qPCR.

To isolate DNA from the filtered water samples to determine fish malacospore release, the filters were cut into smaller pieces using sterilized scissors and homogenized using a tissue lyser (QIAGEN). DNA was then extracted from the lysed filter papers using the DNeasy blood & tissue kit (QIAGEN). DNA was eluted with 100‐µl EB buffer and stored at −20℃ until use in qPCR assays.

qPCR was performed using the *T. bryosalmonae* specific TaqMan probe described in (Bettge, Segner, et al., 2009) and with an applied biosystems 7500 analyser. The qPCR was carried out in a final volume of 20 μl containing 1X TaqMan universal Master Mix (Applied Biosystems), 0.5 μM of each primer (PKDtaqf1: 5′‐GCGAGATTTGTTGCATTTAAAAAG‐3′ and PKDtaqr1: 5′‐GCACATGCAGTGTCCAATCG‐3′), 0.2 μM of the probe PKD (5′‐CAAAATTGTGGAACCGTCCGACTACGA‐3′; 18S rDNA gene of *Tetracapsuloides bryosalmonae*: Accession No. AF190669), 1× of internal control ExoIPC Mix, 1× of IC DNA (TaqMan Univ. MMix w Exog IntPostC, Applied Biosystems) and 2 μl of template DNA (Strepparava et al., 2018). All reactions were carried out in duplicate and no template negative controls were included. PKD infection prevalence (percent *T. bryosalmonae* positive fish sampled per time point) was calculated and individual fish parasite intensities were expressed as DNA copy numbers/g of posterior kidney as described (Strepparava et al., 2018, 2020).

### 2.8 Histopathological examination of fish kidney pathology score (KPS) to assess disease severity

Formalin‐fixed posterior kidney samples were routinely processed for histological examination. One section of 3‐µm thickness was prepared for histopathology and stained with H&E (haematoxylin‐eosin). Histopathological assessment of the kidney pathology score (KPS) was performed to semi‐quantitatively assess disease severity as per previous studies. In short, histopathological alterations of the entire section were scored from 0 (no alterations) to 6 (severe proliferation of the hematopoietic tissue, multiple areas of haemorrhage, multiple areas of necrosis, multiple thrombi, severe multifocal infiltration; Bailey, Rubin, et al., 2018; Bettge, Wahli, et al., 2009; Strepparava et al., 2020). At each time point, the mean value of histological changes for all investigated fish (positive and negative animals for *T. bryosalmonae* DNA tested by qPCR) per replicate and per treatment was calculated and determined as the kidney pathology score (KPS).

### 2.9 Evaluation of fish infection tolerance patterns

Tolerance is defined as the ability of the host to endure the infection. Tolerance as a parameter can be evaluated as the change in host health across different levels of pathogen burden (Råberg, 2014; Regoes et al., 2014). Tolerance curves were generated as per a previous PKD study using brown trout by plotting a proxy for host health, which was determined by PKSI, against parasite intensity determined via qPCR. PKSI is a valid metric for assessing host health as in PKD a swollen kidney results from lymphocyte proliferation, associated granulomatous nephritis and in certain cases increased mortality. From this perspective, PKD in terms of the PKSI provides an ideal host health proxy for examining tolerance relative to other studies that have used less suitable proxies such as growth or survival irrelevant of the disease features. The shallower the slope of the tolerance curve, the more tolerant the host is, the steeper the slope of the curve the less tolerant the host is (Råberg, 2014). Like previous studies, we used the terminology; ‘relative tolerance’ when describing increases or decreases in the experimental groups in comparison to another (Restif & Koella, 2003; Rohr et al., 2010).

### 2.10 RNA isolation and cDNA synthesis

RNA was isolated from the homogenized fraction of the posterior kidney that was stored in TRI‐ reagent^®^, using a Direct‐zol™ RNA MiniPrep w/TRI‐Reagent^®^ kit following the manufacturer's guidelines (Zymo). Prospective traces of genomic DNA contamination were removed with an on‐column DNase treatment provided in the kit. cDNA was synthesized using The GoScript™ Reverse Transcription System following the manufacturer's instructions (Promega). For each sample 1 μg of DNAse free RNA was used. The total volume of the cDNA syntheses was 20 μl which was diluted 1:10 with Nuclease‐Free water (Promega) and stored at −20℃ until RT‐qPCR.

### 2.11 Primer design and RT‐qPCR immune gene expression analysis

To evaluate immune gene transcription levels in the posterior kidney of brown trout, RT‐qPCR was performed with the LightCycler^®^ 96 System (Roche) using SYBR Green PCR core Reagents (Promega) and specific primers either previously optimized or designed using available salmonid sequences (brown trout, Atlantic salmon *Salmo salar* and rainbow trout) obtained from the NCBI database (www.ncbi.nlm.nih.gov) based on Primer3 algorithms and adjusted manually if necessary (Koressaar & Remm, 2007; Untergasser et al., 2012; Table S1). In our previous work several reference genes were tested in brown trout tissues and *ef‐1α* (elongation factor‐1α) was identified as a stable reference transcript in the posterior kidney of both control and fish infected with *T. bryosalmonae* at differing levels of PKD pathogenesis, thus this gene was taken as reference to which all target genes were related (Bailey, Strepparava, et al., 2019). All samples were measured in duplicate. The total reaction volume was 20 µl, containing 10 µl GoTaq^®^ qPCR Master Mix, 1 µl of 10 µM primer stocks (final concentration 500 nM), 3 µl Nuclease‐Free water and 5 μl of the diluted cDNA synthesis mix. RT‐qPCR was performed using the following settings: 5 min 95℃, followed by 40 cycles of amplification. Each cycle consisted of 3 s of denaturation at 95℃, annealing and elongation at 60℃ for 30 s. The PCR was terminated with a melting curve analysis starting with a denaturation step of 95℃ followed by the start ramping temperature of 60℃ for 30 s. At first the data were analysed with the Roche light cycler 96 Application Software Version 1.1 then data were evaluated according to the 2−^ΔΔCt^ method. ‘No template negative controls’ and *minus* reverse transcriptase controls were included in all assays.

### 2.12 Statistical analysis

To investigate *T. bryosalmonae* infection in different age phenotypes we statistically evaluated YOY control, YOY infected, juvenile 1+ control and juvenile 1+ infected. To compare if host‐infection life history‐modulated PKD outcome we evaluated CI, PI, RI and RCI response phenotypes using juvenile 1+ control fish as an absolute control. In both sets of evaluation groups, we compared mortality over the course of an experiment using a Kaplan–Meier analysis to generate survival curves and a log‐rank test to examine the impact of infection on mortality. While PKD infection prevalence in the infected fish was assessed using a binomial generalized linear model (GLM), due to the data being proportional (% of fish infected at each time point). Differences in SGR, parasite intensity in the posterior kidney, fish malacospore release, disease severity (KPS) and immune gene transcripts measured in the posterior kidney were tested for either using the Student's *t* test or using a one‐way ANOVA and significant differences revealed with Tukey's post‐hoc test. Any data failing normality tests (Kolmogorov–Smirnov test) and displaying heterogeneity of variance were tested (statistically), applying the nonparametric Kruskal–Wallis ANOVA on ranks, and Dunn's nonparametric multiple comparison tests to reveal differences. Tolerance was quantified by correlating host health with parasite intensity in the posterior kidney and evaluated by the variation of the slope. These data were fit with a simple linear regression model in Prism after testing several other nonlinear models and the relationship between the two variables tested statistically as per our earlier study (Bailey, Strepparava, et al., 2019). Gene transcript data were tested at the ΔCt stage before the log transformation. Infection group data were normalized to unexposed controls of the same age, that is, YOY control fish to YOY infection fish and juvenile 1+ infection, CI, PI, RCI and RI to juvenile 1+ control fish. We did not adjust for the declining tank population during fish malacospore release between groups (due to fish sampling) due to the differences in infection prevalence. All statistical analyses were performed using R (version 3.1.0; de Micheaux et al., 2013; R Development Core Team, 2014) or SigmaPlot 12.0 (Systat Software) and graphically presented with GraphPad Prism 6 (GraphPad Software, Inc.). Significance was set at *p* ≤ 0.05.

## 3 RESULTS

### 3.1 Parasite‐centric parameters—*T. bryosalmonae* infection prevalence

**FIGURE 3:**
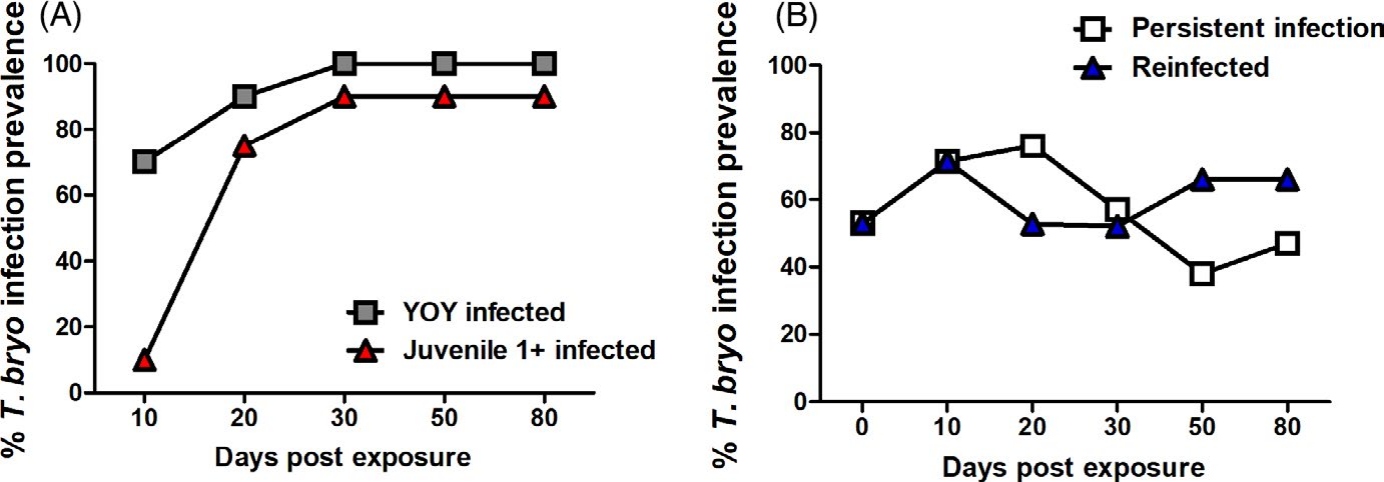
*Tetracapsuloides bryosalmonae* infection prevalence of (A) young‐of‐the‐year (YOY) and juvenile 1+ infected fish and in (B) persistent infection (PI) and re‐infection (RI) fish. Prevalence was determined by measurement of *T. bryosalmonae* in the posterior kidney. Prevalence estimates at each time point were based on *n* = 20 for (A) and fluctuates between 15 and 21 for (B)

Overall *T. bryosalmonae* infection prevalence (% of infected fish sampled per time point) was significantly greater in YOY infected fish (reaching a maximum of 100%) relative to juvenile 1+ infected fish (reaching a maximum of 90%; Figure 3A). There were no significant differences when comparing infection prevalence in the re‐exposure treatments and prevalence levels remained comparable throughout. For example, the overall mean infection prevalence was: 58% for the PI and 61% for the RI fish (Figure 3B).

### 3.2 *T. bryosalmonae* intensity and proliferation

**FIGURE 4:**
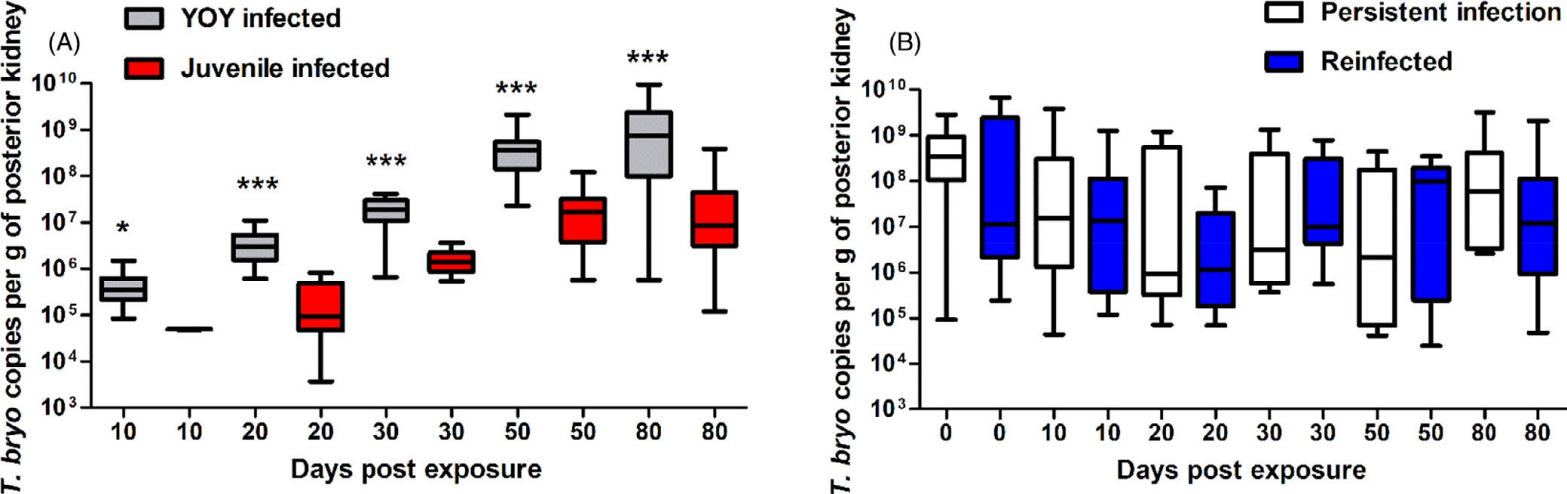
Parasite intensity of (A) young‐of‐the‐year (YOY) and juvenile 1+ infected fish and (B) fish with a persistent infection (PI) and re‐infected (RI) fish (Median ± *SE*). Parasite intensity was expressed as 18S rRNA gene copies of *Tetracapsuloides bryosalmonae* standardised to posterior kidney weight. Amount of asterisk indicates level of significance (*p* < 0.05*, *p* < 0.005** and *p* < 0.001***) when parasite intensity at each time point is compared between treatments. *n* = 20 fish were sampled at each time point for (A) and fluctuates between 15 and 21 for (B)

The concentration of *T. bryosalmonae* DNA per gram of posterior kidney tissue was used to estimate parasite intensity in infected fish. In the YOY and juvenile 1+ infected fish, parasite intensity steadily increased over the course of the experiment plateauing at 80 dpe. In this setting, there was significant greater parasite burden in the YOY infected fish relative to the juvenile 1+ infected fish at every time point investigated [Figure 4A; 10 dpe (*p* = 0.032), 20 dpe (*p* < 0.001), 30 dpe (*p* < 0.001), 50 dpe (*p* < 0.001), 80 dpe (*p* < 0.001)]. By contrast, in the re‐exposed fish the mean of parasite intensity tended to stay constant in both infected response groups (PI and RI) and no significant differences were found between the treatments at any time point (Figure 4B).

### 3.3 Malacospore release from fish

**FIGURE 5:**
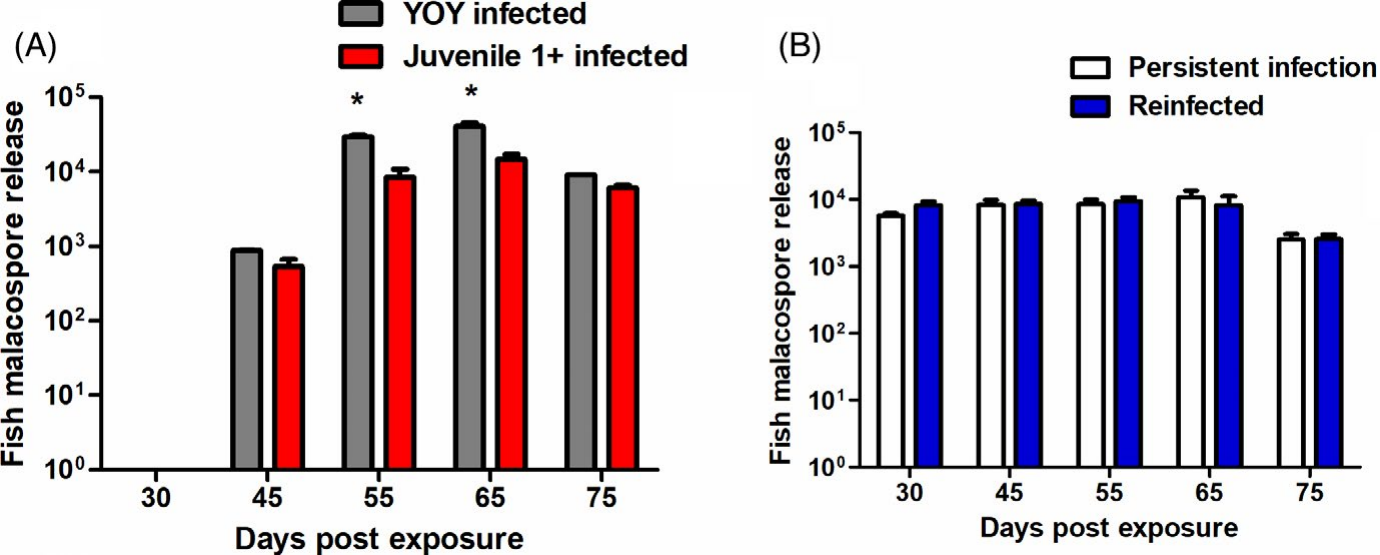
Fish malacospore release in tanks consisting of (A) young‐of‐the‐year (YOY) and juvenile 1+ infected fish and (B) fish with a persistent infection (PI) and re‐infected (RI) fish measured as parasite intensity per litre of water (averaged between treatment tanks; *M* ± *SE*) at different time points. Asterisk above data point indicates significant increase relative to other experimental group at the same time point and the amount of asterisk indicates level of significant increase (*p* < 0.05*, *p* < 0.005** and *p* < 0.001***)

The onset of fish malacospore release was investigated from 30 dpe until 75 dpe. We chose this time point as previously we had found infected fish had started fish malacospore release at 45 dpe (Bailey, Strepparava, et al., 2019; Strepparava et al., 2020). Thus, we wanted to see if an earlier onset occurred. Although this did not transpire in either experimental treatment in the fish of different ages infected for the first time (YOY infected and juvenile 1+ infected). However, fish malacospore release did begin at 45 dpe which agreed with our previous studies. The temporal trend of fish malacospore release was similar in both groups increasing and peaking at 65 dpe before decreasing at 75 dpe. Furthermore, the temporal increase was significantly greater in the YOY infected fish at 55 (*p* = 0.043) and 65 dpe relative to the juvenile 1+ infected fish (*p* = 0.045; Figure 5A).

In the re‐exposure experimental groups in both the PI and the RI groups the malacospore release from fish were detected at the first time point investigated (30 dpe), suggesting the consistent release of fish malacospores from persistently infected fish occurs regardless of re‐exposure. The number of fish malacospore release was comparable between the treatments at every time point and there were no significant differences between the amounts of fish malacospore release at each time point. The number of fish malacospore release appeared to decline in both the PI and RI groups at 75 dpe as per the YOY and juvenile 1+ infected groups (Figure 5B).

### 3.4 Host‐centric parameters—Mortality and SGR

Over the course of the experiment (80 dpe) there was only low mortality in all experimental treatments. However, there was a decrease of survival in YOY‐infected fish which was significant when compared to juvenile 1+ infected fish (*p* = 0.01). No significant differences were found when comparing survival in PI fish or RI fish, thus re‐exposure did not have any effects on mortality (Figure S1).

**FIGURE 6:**
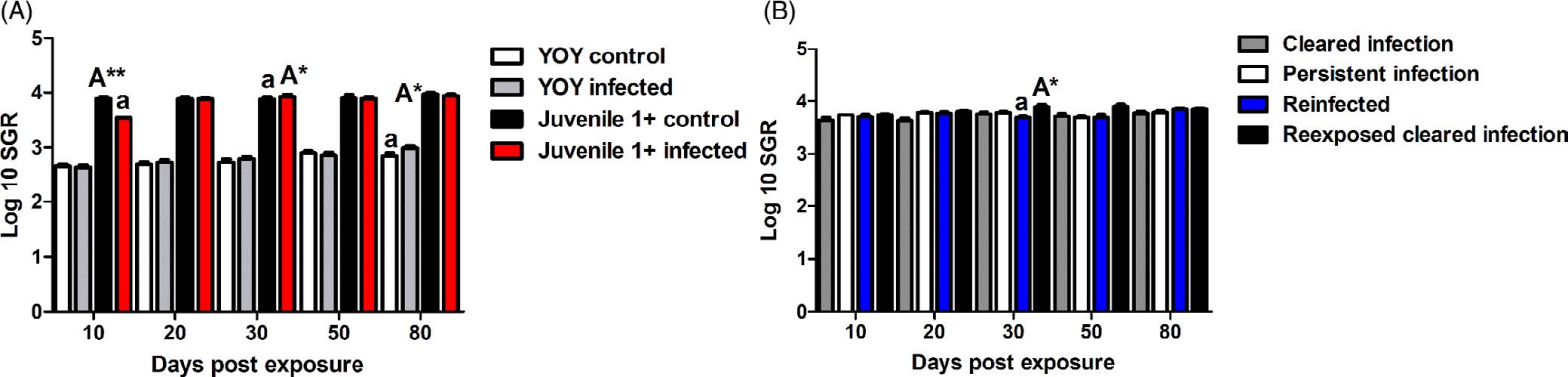
Specific growth rate (SGR) of (A) fish of different age classes, young‐of‐the‐year (YOY) and juvenile 1+ fish control and infection treatments and (B) juvenile fish either re‐exposed [re‐infected (RI) and RCI] or not re‐exposed [cleared infection (CI) and persistent infection (PI); *M* ± *SE*] at different time points. Letters indicate significant differences; A indicates a significant increase to corresponding time point with a lower case a. Amount of asterisk indicates level of significant (*p* < 0.05*, *p* < 0.005** and *p* < 0.001***). For A; *n* = 20 per time point, for B; *n* ≥ 6 per time point

Specific growth rate is classically used as a proxy to assess the growth of aquatic organisms under experimental conditions using specimen weight, thus we were interested to see if parasite infection impacted growth. Concerning the comparison of the SGR in fish with different age phenotypes (YOY control, YOY infected, juvenile 1+ control, juvenile 1+ infected) there was a significant increase in the SGR of YOY infected fish relative to the YOY control fish at 80 dpe (*p* = 0.04; Figure 6A). Regarding the juvenile 1+ fish, there was a significantly greater SGR in the juvenile 1+ control fish at 10 dpe in comparison to the juvenile 1+ infected fish (*p* < 0.01). However, at 30 dpe this was reversed as there was a significant increase in the SGR of juvenile 1+ infected fish in comparison to the juvenile 1+ control fish (*p* = 0.02). In the heterogeneous host infection life‐history groups (CI, PI, RCI and RI) at the end of the experiment at 80 dpe the SGR of all the experimental fish was comparable. Nonetheless, there was a significant difference at 20 dpe between the SGR of the treatments using a one‐way ANOVA, but the post‐hoc comparison was not significant (Figure 6B). While at 30 dpe, there was a significant increase in the SGR of the RCI fish in comparison to the RI fish.

### 3.5 Host disease severity

**FIGURE 7:**
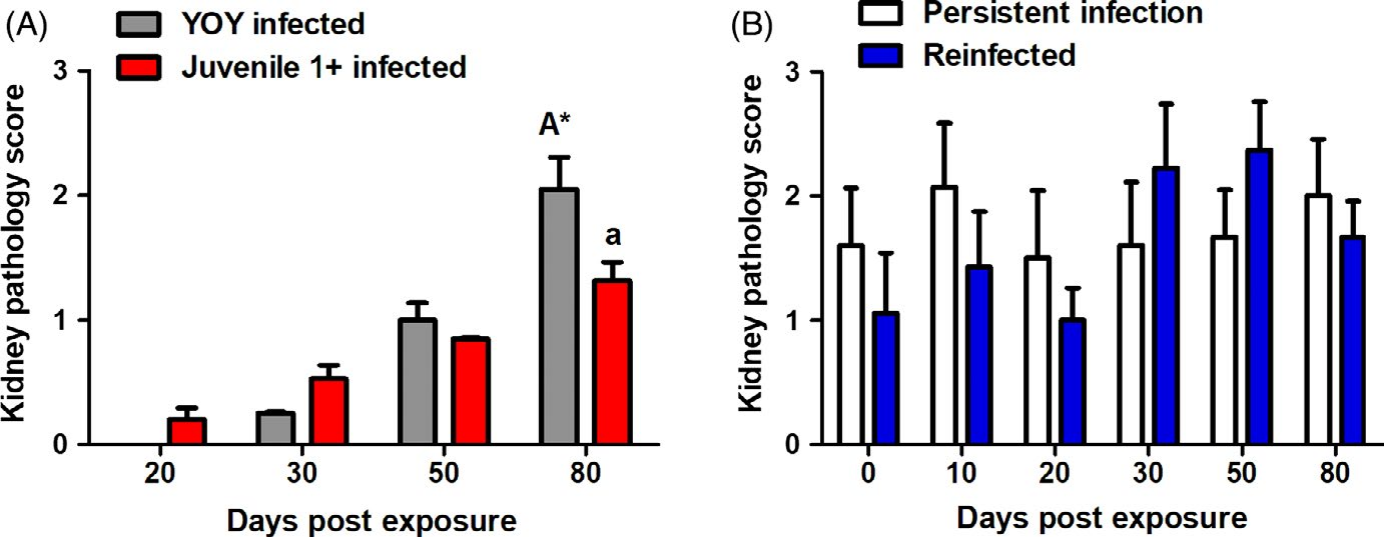
Kidney pathology score (KPS) in (A) young‐of‐the‐year (YOY) and juvenile 1+ infected fish and (B) persistent infection (PI) and re‐infected (RI) fish. Amount of asterisk indicates level of significant (*p* < 0.05*, *p* < 0.005** and *p* < 0.001***) when time points are compared between treatments. *n* = 20 fish were sampled at each time point for (A) and >6 for (B)

Fish from each experimental group infected with *T. bryosalmonae* showed symptoms consistent with those described for *T. bryosalmonae*‐infected brown trout (Bailey, Rubin, et al., 2018). These developments in pathology must be attributed exclusively to the infection by *T. bryosalmonae*, as no other pathogenic agents were found. Histological evaluation of control fish revealed no signs of PKD, as an outcome, the KPS for YOY and juvenile 1+ control fish evaluated was a 0 score in every fish examined. The KPS in YOY and juvenile 1+ infected fish increased temporally in both treatments. This increase in KPS was significantly greater in the younger fish reaching 2.05 in the YOY infected fish and only 1.3 in juvenile 1+ infected fish at 80 dpe (*p* = 0.02; Figure 7A).

In the re‐exposure experiment, in fish without the presence of *T. bryosalmonae* determined via qPCR (CI and RCI treatments) most showed no pathological alterations in the posterior kidney, and neither were parasites observed. Nevertheless, five fish in the entire study (CI = 2, RCI = 3) were found with minimal infrequent parasite‐induced alterations, although as mentioned, in these fish no parasites were seen histologically or detected via qPCR. Regarding fish with parasite burdens, the PI and RI fish, the KPS had no strong temporal patterns similar to infection prevalence and parasite intensity data, and no significant differences were observed at any time point (Figure 7B).

### 3.6 Host infection tolerance

**FIGURE 8:**
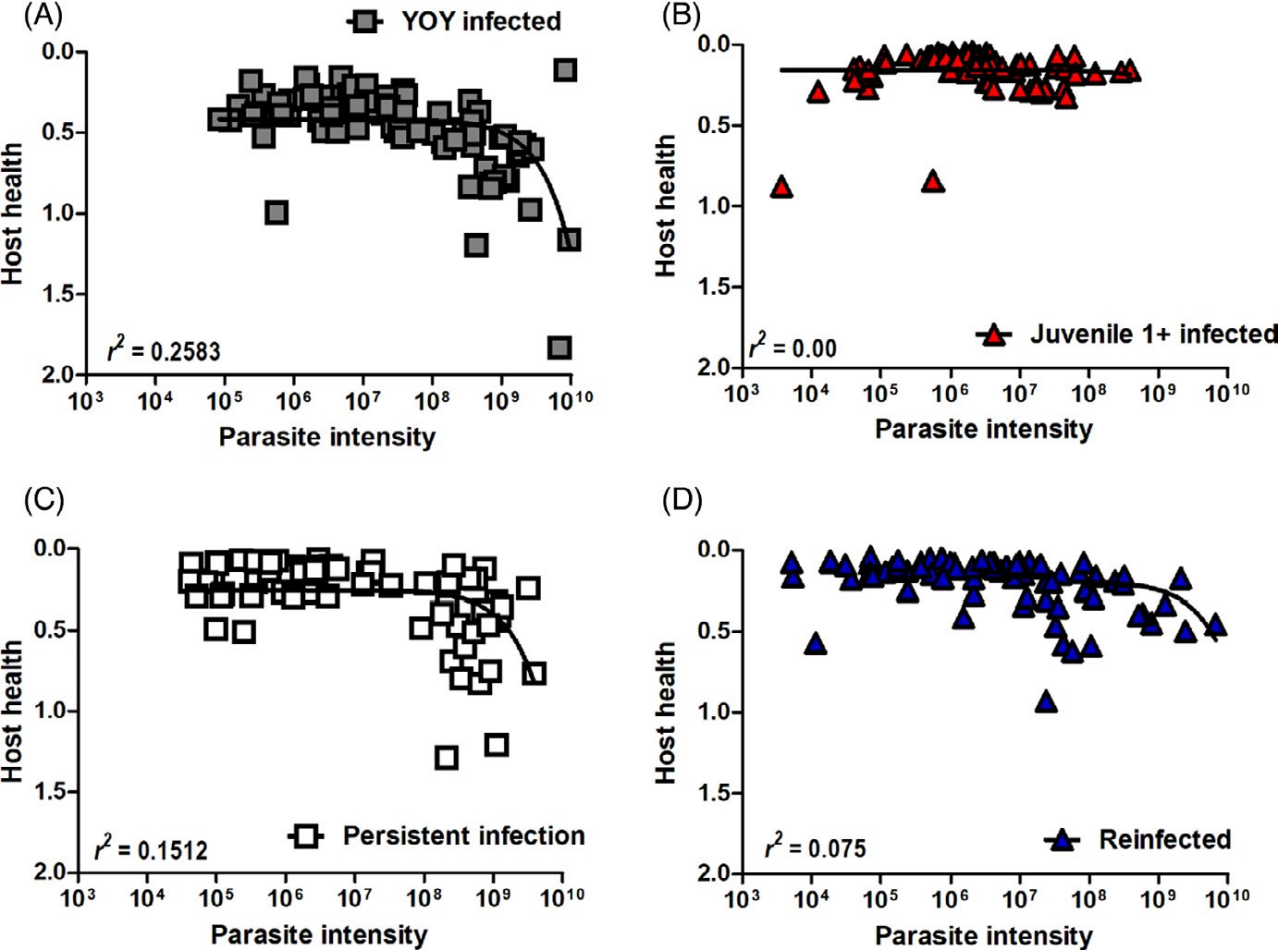
Quantifying host patterns of PKD tolerance. Tolerance curves of (A) young‐of‐the‐year (YOY) infected fish (B) juvenile 1+ infected fish (C) persistent infection (PI) fish and (D) re‐infected (RI) fish. *n* fluctuates between 68 and 93 for reach treatment

Tolerance was quantified by correlating host health (via the PKSI) with parasite intensity and evaluated by the variation of the slope. The host health of YOY infected fish decreased more drastically with lower parasite intensity in comparison to juvenile 1+ infected fish. In the YOY infected fish, the relationship between these two variables was significant (*p* < 0.001; Figure 8A,B). Therefore, indicating that juvenile 1+ *T. bryosalmonae*‐infected brown trout have increased relative tolerance during PKD in comparison to YOY infected fish, as their host health does not deteriorate in the same fashion. In the PI and RI fish, relative tolerance was comparable, and in both these groups, there was a significant relationship between the two factors [PI (*p* < 0.001), RI (*p* < 0.012; Figure 8C,D)].

### 3.7 Host regulation of immune gene transcripts

Due to the extensive results regarding the regulation of immune gene transcripts in hosts with different age classes and in hosts with heterogeneous infection life histories these results are reported separately.

#### 3.7.1 Hosts with different age classes

**FIGURE 9:**
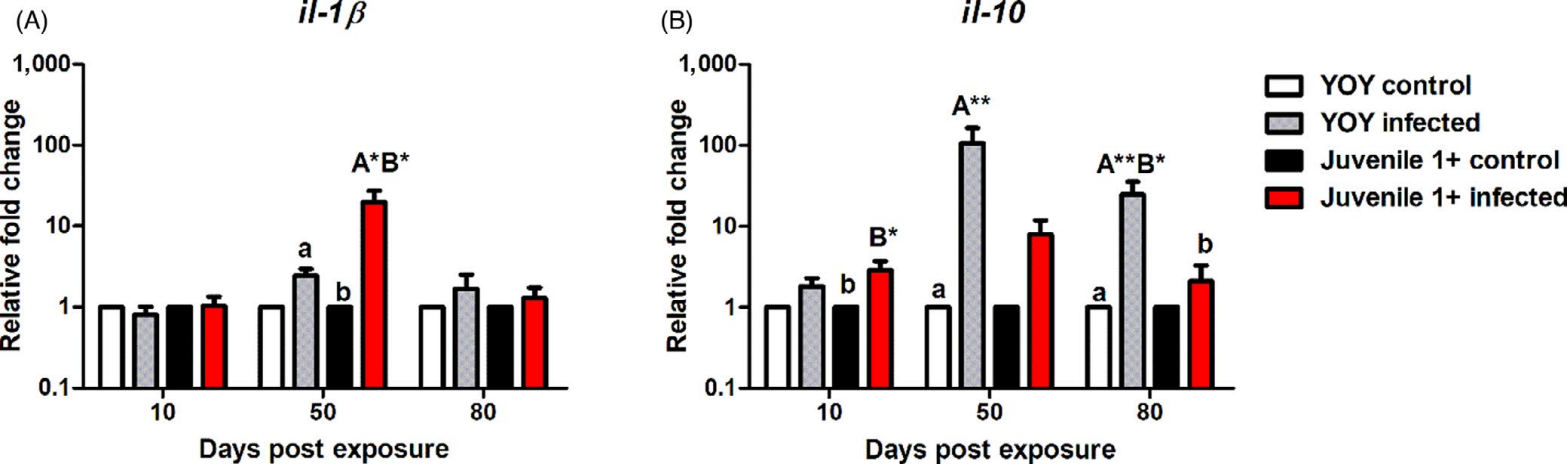
Regulation of immune gene transcripts. Relative fold change of (A) *il‐1β* and (B) *il‐10* measured in the posterior kidney of *Tetracapsuloides bryosalmonae*‐infected brown of different age classes at days post exposure (Arithmetic Mean ± *SE*). Relative fold change was normalized to brown trout reference gene *ef‐1α* and subsequently expressed as fold change relative to expression levels of specific control fish. Significant differences exist are indicated with capital and lower (for instance ‘A’ indicates a significant difference relative to ‘a’, and ‘B’ indicates a significant difference relative to ‘b’). Number of asterisks indicates level of significance (*p* < 0.05*, *p* < 0.005** and *p* < 0.001***). *n* = 6 per time point, per control and infection treatments

In *T. bryosalmonae*‐infected fish from different age classes in both the YOY and juvenile 1+ infected fish, *il‐1β* was unresponsive at the earliest measured time point (10 dpe), before becoming only mildly upregulated in the YOY infected fish at 50 and 80 dpe. However, at 50 dpe in juvenile 1+ infected fish the mRNA levels of *il‐1β* were significantly elevated relative to both uninfected fish of the same age (*p* = 0.02) and of YOY infected fish (*p* = 0.03; Figure 9A). Concerning the regulation of *il‐10*, in YOY infected fish, *il‐10* was significantly upregulated (105‐fold change (fc) at 50 dpe relative to YOY control fish (*p* = 0.002). Likewise, at 80 dpe in YOY infected fish this gene was also significantly upregulated in comparison to control fish (*p* = 0.002) and to juvenile 1+ infected fish (*p* = 0.013). In contrast, the gene was significantly upregulated in juvenile 1+ infected fish at 10 dpe in comparison to juvenile 1+ control fish (*p* = 0.034; Figure 9B).

##### Cell‐mediated immunity: T cell transcripts

**FIGURE 10:**
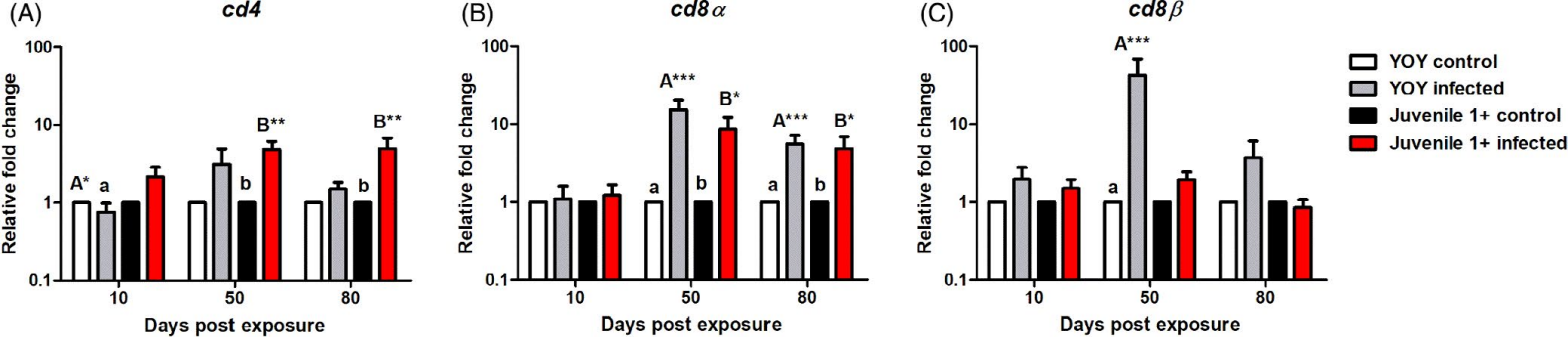
Regulation of T cell transcripts. Relative fold change of (A) *cd4*; (B) *cd8α* and (C) *cd8β* measured in the posterior kidney of *Tetracapsuloides bryosalmonae*‐infected brown trout of different age classes at days post exposure (Arithmetic Mean ± *SE*). Relative fold change was normalized to brown trout reference gene *ef‐1α* and subsequently expressed as fold change relative to expression levels of specific control fish. Significant differences exist are indicated with capital and lower (for instance ‘A’ indicates a significant difference relative to ‘a’, and ‘B’ indicates a significant difference relative to ‘b’). Number of asterisks indicates level of significance (*p* < 0.05*, *p* < 0.005** and *p* < 0.001***). *n* = 6 per time point, per control and infection treatments

To get an overview of the T cell response we measured a general panel of T cell markers; *cd4*, *cd8α* and *cd8β*. In YOY infected fish, *cd4* was significantly downregulated at 10 dpe relative to the control (*p* = 0.04) before increasing to a mild level of upregulation at 50 and 80 dpe. In juvenile 1+ infected fish, *cd4* was upregulated at every time point. Specifically, in these fish, this transcript was significantly upregulated relative to juvenile 1+ control fish at 50 (*p* = 0.003) and 80 dpe (*p* = 0.003; Figure 10A). The regulation pattern of *cd8α* transcripts was comparable in both infection groups: ≈1 fold change (fc) at 10 dpe, before increasing at 50 dpe (YOY (*p* < 0.001) juvenile 1+ (*p* < 0.01) and slightly declining at 80 dpe in both YOY (*p* < 0.001) and juvenile 1+ infected fish (*p* = 0.02), with significant upregulations in comparison to their respective controls at these latter time points in both infection groups (Figure 10B). Concerning *cd8β* transcripts, in YOY infected fish, *cd8β* was upregulated at every time point being strongly significantly upregulated at 50 dpe relative to the YOY control (*p* < 0.001). On the other hand, this gene was only mildly expressed in juvenile 1+ infected fish and even downregulated at 80 dpe relative to the juvenile 1+ control (Figure 10C).

##### Humoral immunity: *blimp1* and immunoglobulin transcripts

We measured the three fish Igs, *igm*, *igt* and *igd*, in terms of both the membrane and secreted transcripts as well as *blimp1*. Regarding the mRNA expression levels of *blimp1*, in YOY infected fish the gene was upregulated at all time points, increasing in a temporal fashion. In this context, in YOY infected fish, *blimp1* was strongly significantly expressed at 50 dpe relative to control fish (*p* < 0.001) and at 80 dpe relative to both control (*p* < 0.001) and juvenile 1+ infected fish (*p* = 0.006). Alternatively, in juvenile 1+ infected fish, the transcript was only transiently significantly upregulated at 10 dpe (*p* < 0.01) before becoming lowly expressed for the remainder of the experiment (1.56 fc at 50 dpe and 1.03 fc at 80 dpe; Figure 9A).

**FIGURE 11:**
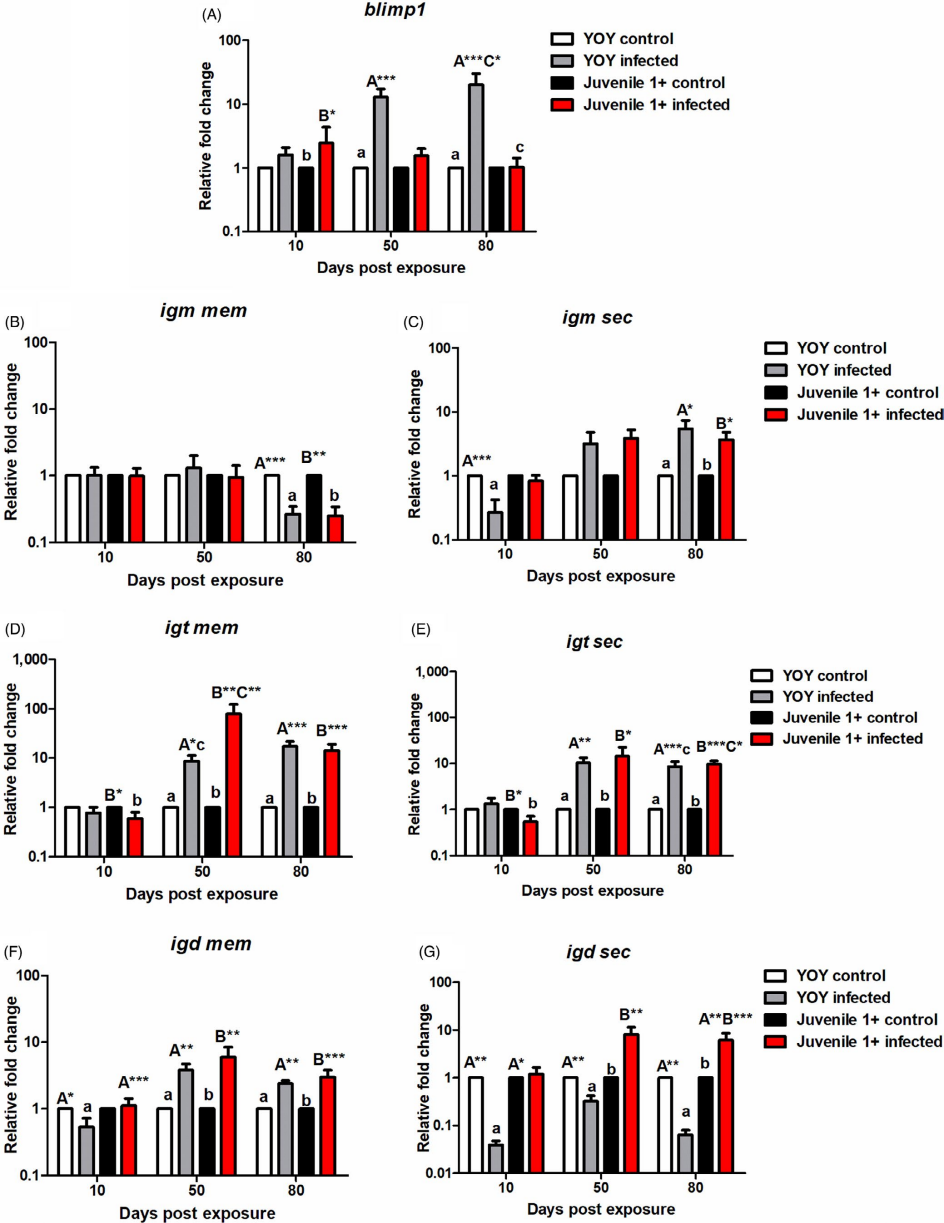
Regulation of B cell and immunoglobulin transcripts. Relative fold change of (A) *blimp1*; (B) *igm mem*; (C) *igm sec*; (D) *igt mem*; (E) *igt sec*; (F) *igd mem* and (G) *igd sec* measured in the posterior kidney of *Tetracapsuloides bryosalmonae*‐infected brown trout of different age classes at days post exposure (Arithmetic Mean ± *SE*). Relative fold change was normalized to brown trout reference gene *ef‐1α* and subsequently expressed as fold change relative to expression levels of specific control fish. Significant differences exist are indicated with capital and lower (for instance ‘A’ indicates a significant difference relative to ‘a’, and ‘B’ indicates a significant difference relative to ‘b’). Number of asterisks indicates level of significance (*p* < 0.05*, *p* < 0.005** and *p* < 0.001***). *n* = 6 per time point, per control and infection treatments

*igm mem* was generally unresponsive in both infection groups and significantly downregulated relative to the specific control in both infection groups at 80 dpe [YOY (*p* < 0.001), juvenile 1+ (*p* = 0.002); Figure 11B]. While mRNA levels of *igm sec* followed a similar pattern in both infection groups, in contrast the transcript level became significantly upregulated as the experiment progressed. From this perspective, *igm sec* was downregulated at 10 dpe in both infection groups (but only significantly in YOY infected fish (*p* = 0.001). Then as PKD pathogenesis developed the transcript was significantly upregulated in both the infection groups at 80 dpe relative to their respective controls [YOY (*p* < 0.01), juvenile 1+ (*p* < 0.01; Figure 11C].

Both membrane bound and secretory forms of *igt* also appeared to increase temporally in the infection groups. As a case in point, *igt* mem expression levels were initially downregulated at 10 dpe in both infection groups, even being significantly inhibited in juvenile 1+ infected fish (*p* = 0.024). Then at 50 dpe, in both infection treatments *igt* mem expression increased, becoming significantly elevated in YOY infected fish versus YOY control fish (*p* < 0.01), and strongly significantly upregulated (78.89 fc) in juvenile 1+ infected fish versus juvenile 1+ control fish (*p* = 0.002) and YOY infected fish (*p* < 0.004). Furthermore, in comparison to their respective controls, both infection groups had significantly elevated mRNA levels of *igt* mem at 80 dpe [YOY (*p* < 0.001), juvenile 1+ (*p* < 0.001); Figure 11D]. Concerning *igt sec* expression, at 10 dpe in YOY infected fish this transcript was only lowly upregulated although at the same time point in juvenile 1+ infected fish, *igt sec* was significantly downregulated relative to the juvenile 1+ control (*p* = 0.022). Nevertheless, comparable to *igt* mem transcripts, the mRNA levels eventually increased with time and likely disease pathogenesis. In this context, *igt sec* was significantly upregulated in both infection groups relative to their controls at 50 dpe [YOY (*p* < 0.001), juvenile 1+ (*p* < 0.02)] and 80 dpe [YOY (*p* < 0.001), juvenile 1+ (*p* < 0.001)] and in juvenile 1+ infected group at 80 dpe relative to the YOY infected group (*p* < 0.04; Figure 11E).

*igd* mem was significantly downregulated at 10 dpe in YOY infected fish relative to the control (*p* < 0.03) and juvenile 1+ infected fish (*p* < 0.001), before increasing at 50 (*p* = 0.004) and 80 dpe (*p* = 0.004) becoming significantly upregulated in comparison to the YOY control. Likewise, a significant increase in the expression of *igd* membrane transcripts versus the control also transpired in juvenile 1+ infected fish at 50 (*p* = 0.002) and 80 dpe (*p* < 0.001; Figure 11F). Alternatively, *igd sec* transcripts were surprisingly significantly downregulated at every time point in the YOY infected fish, for example, at 10 dpe versus YOY control (*p* = 0.002) and juvenile 1+ infected (*p* = 0.031); at 50 dpe versus YOY control (*p* = 0.005) and at 80 dpe versus YOY control (*p* = 0.004) and juvenile 1+ infected fish (*p* < 0.001). On the contrary, in the juvenile 1+ infected fish there was significant elevation of this transcript in comparison to juvenile 1+ control fish at 50 dpe (*p* = 0.005) and at 80 dpe (*p* < 0.001; Figure 11G).

#### 3.7.2 Regulation of immune gene transcripts in hosts with heterogeneous infection life histories

**FIGURE 12:**
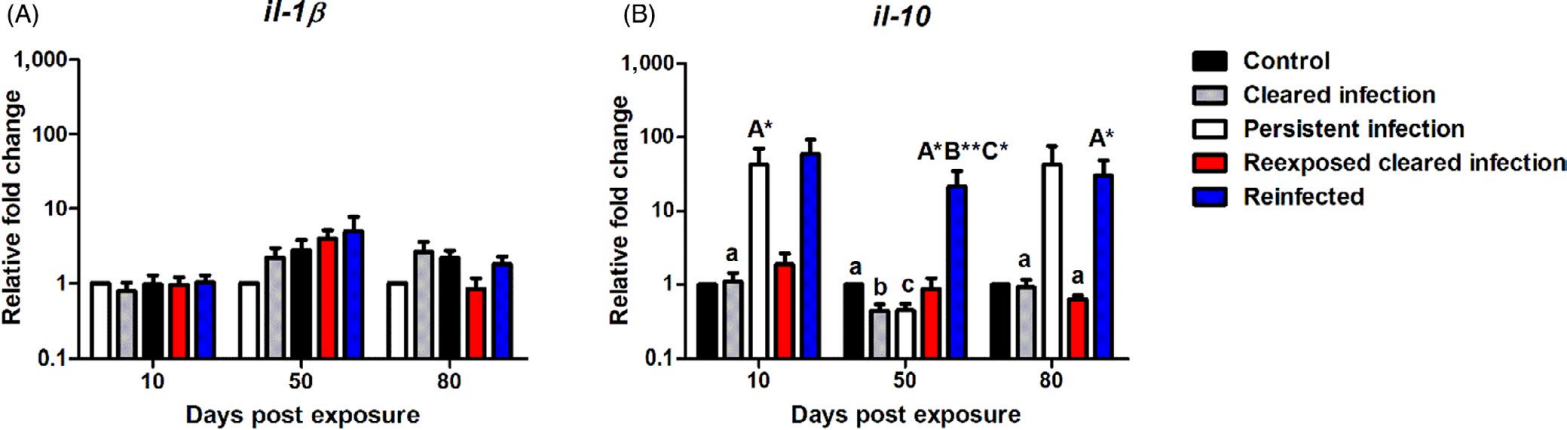
Regulation of immune gene transcripts. Relative fold change of (A) *il‐1β* and (B) *il‐10* measured in the posterior kidney of *Tetracapsuloides bryosalmonae*‐infected brown trout with different infection histories at days post exposure (Arithmetic Mean ± *SE*). Relative fold change was normalized to brown trout reference gene *ef‐1α* and subsequently expressed as fold change relative to expression levels of juvenile 1+ control fish. Significant differences are indicated with capital and lower cases (for instance ‘A’ indicates a significant difference relative to ‘a’, and ‘B’ indicates a significant difference relative to ‘b’). Number of asterisks indicates level of significance (*p* < 0.05*, *p* < 0.005** and *p* < 0.001***). *n* = 6 per time point, per control and infection treatments

In the evaluation of fish with different infection life histories (CI, PI, RCI, RI) regarding the expression of the classic pro inflammatory cytokine *il‐1β*, no significant differences were found between any of the treatment groups at any time point (Figure 12A). Pertaining to the regulation of *il‐10*, this transcript was strongly expressed in both the PI and RI infected fish (Figure 12B). In this fashion, in PI fish there was significant elevation relative to CI fish at 10 dpe (*p* = 0.013). While in RI fish, *il‐10* was expressed at >20 fc at every time point, resulting in significantly greater mRNA levels at 50 dpe in RI fish compared to all non‐parasitized treatments [control (*p* < 0.02), CI (*p* = 0.004), RCI (*p* < 0.014)] and at 80 dpe versus CI (*p* < 0.02) and RCI fish (*p* < 0.03).

##### Cell‐mediated immunity: T cell transcripts

**FIGURE 13:**
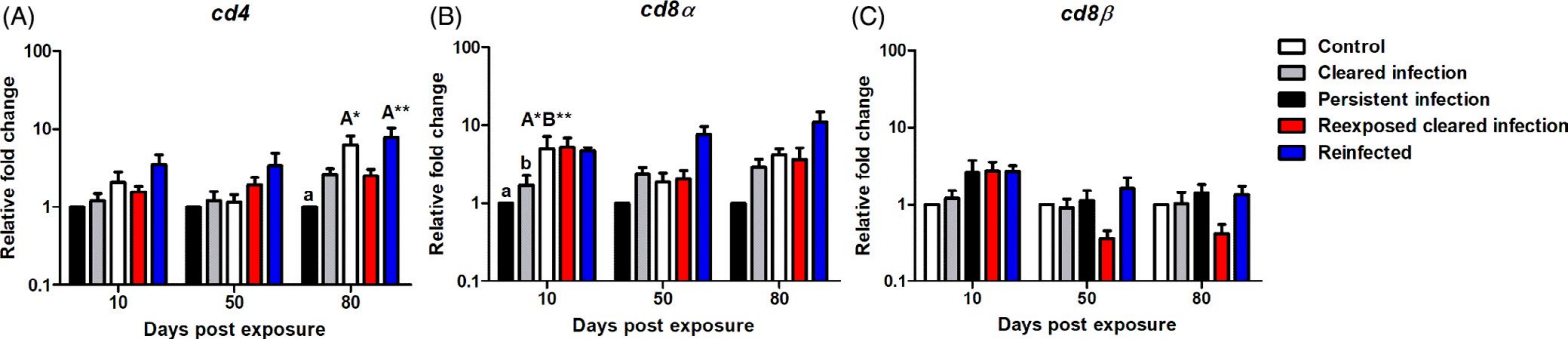
Regulation of T cell transcripts. Relative fold change of (A) *cd4*; (B) *cd8α* and (C) *cd8β* measured in the posterior kidney of *Tetracapsuloides bryosalmonae*‐infected brown trout with different infection histories at days post exposure (Arithmetic Mean ± *SE*). Relative fold change was normalized to brown trout reference gene *ef‐1α* and subsequently expressed as fold change relative to expression levels of juvenile 1+ control fish. Significant differences exist are indicated with capital and lower cases (for instance ‘A’ indicates a significant difference relative to ‘a’, and ‘B’ indicates a significant difference relative to ‘b’). Number of asterisks indicates level of significance (*p* < 0.05*, *p* < 0.005** and *p* < 0.001***). *n* = 6 per time point, per control and infection treatments

*cd4* transcripts were only moderately expressed in all experimental groups with a trend for upregulation to be slightly stronger in the PI and RI fish. In this setting, at 80 dpe, there was significant elevation of *cd4* mRNA levels in both infected fish groups [PI (*p* = 0.002) and RI (*p* < 0.01)] in comparison to the unexposed control (Figure 13A). *cd8α* was significantly upregulated in PI at 10 dpe relative to the control (*p* < 0.012) and the CI fish (*p* < 0.002). At later time points, there was a greater expression of this gene in the RI fish relative to the other treatments although this upregulation was not significant (Figure 13B). No significant differences were found between any treatment at any time points concerning *cd8β* (Figure 13C).

##### Humoral immunity: *blimp1* and immunoglobulin transcripts

**FIGURE 14:**
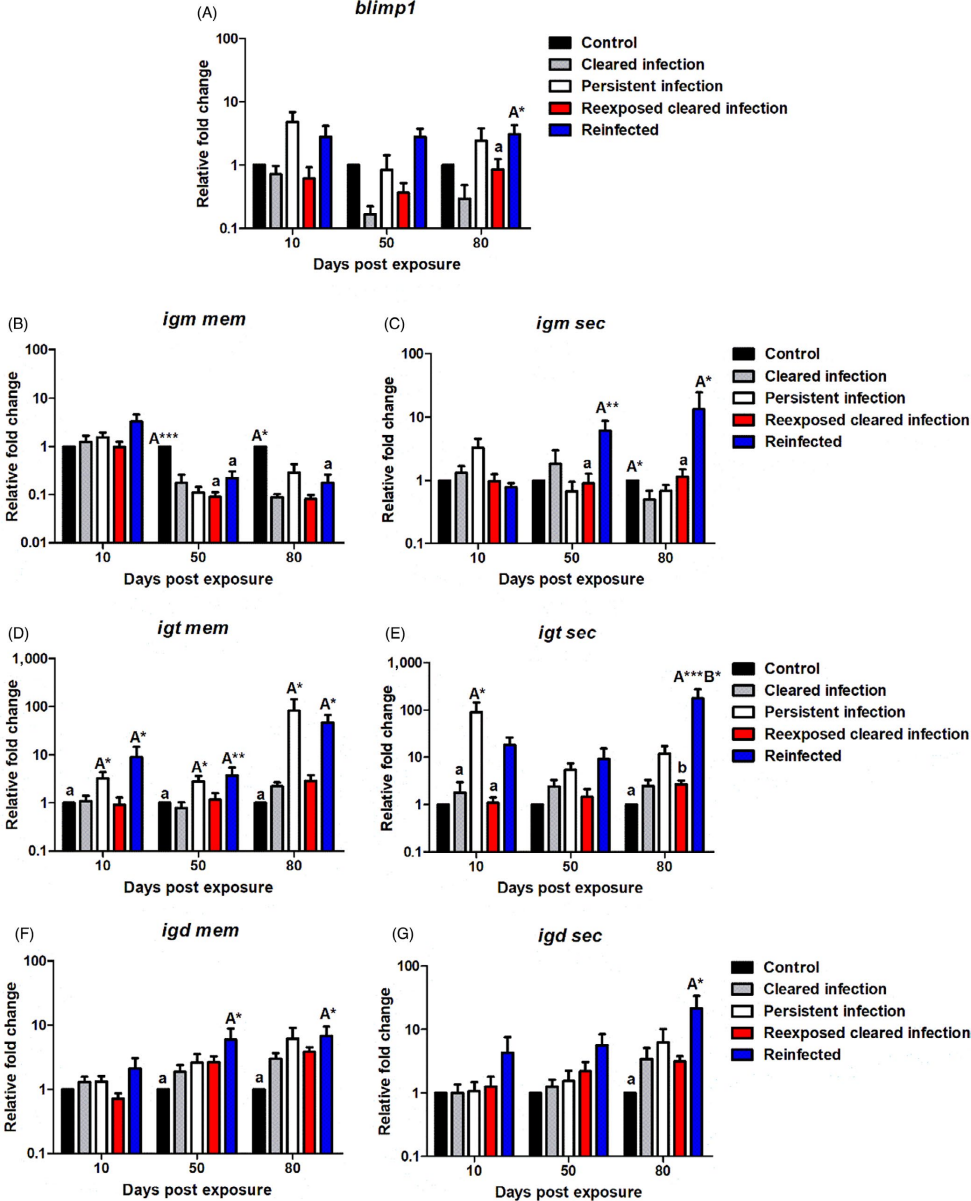
Regulation of B cell and immunoglobulin transcripts. Relative fold change of (A) *blimp1*; (B) *igm mem*; (C) *igm sec*; (D) *igt mem*; (E) *igt sec*; (F) *igd mem* and (G) *igd sec* measured in the posterior kidney of *Tetracapsuloides bryosalmonae*‐infected brown trout with different infection histories at days post exposure (Arithmetic Mean ± *SE*). Relative fold change was normalized to brown trout reference gene *ef‐1α* and subsequently expressed as fold change relative to expression levels of juvenile 1+ control fish. Significant differences exist are indicated with capital and lower cases (for instance ‘A’ indicates a significant difference relative to ‘a’, and ‘B’ indicates a significant difference relative to ‘b’). Number of asterisks indicates level of significance (*p* < 0.05*, *p* < 0.005** and *p* < 0.001***). *n* = 6 per time point, per control and infection treatments

*blimp1* was more strongly expressed in both infected fish (PI and RI) being moderately upregulated at every time point in comparison to the uninfected fish. In addition, *blimp1* was significantly upregulated in RI fish relative to RCI fish at 80 dpe (*p* = 0.014; Figure 14A). In the same fashion as the infected fish with different age classes *igm mem* was generally unresponsive or downregulated, with the only exemption being a mild upregulation of *igm mem* at 10 dpe in the RI fish (3.33 fc; Figure 14B). Moreover, in the RCI and RI fish, *igm mem* expression was significantly lower relative to the control at 50 dpe both (*p* < 0.001) and at 80 dpe specifically in the RI fish relative to the control (*p* = 0.014). On the contrary, *igm sec* was significantly upregulated in the RI fish at 50 dpe versus RCI (*p* = 0.004) fish and at 80 dpe versus RCI fish (*p* < 0.05). In the RCI fish also at 80 dpe, the transcript was significantly downregulated relative to the control (*p* < 0.05; Figure 14C).

Concerning *igt* transcript levels, both membrane and secretory bound forms were generally upregulated in fish with presence of parasites (PI and RI fish). From this perspective, there was a significantly greater expression of *igt mem* transcripts in PI and RI fish relative to the control fish at every time point investigated [PI: 10 dpe (*p* = 0.01), 50 dpe (*p* < 0.03), 80 dpe (*p* < 0.05, RI: 10 dpe (*p* = 0.01), 30 dpe (*p* = 0.004), 50 dpe (*p* < 0.01)] with the strongest expression in both groups coming at 80 dpe (PI: 83.11 fc and RI: 46.54 fc; Figure 14D). Likewise, for *igt sec* regulation there was a significant strong upregulation in the PI fish at 10 dpe [vs. CI (*p* = 0.02) and RCI fish (*p* = 0.02)] and for the RI fish at 80 dpe [(vs. the control (*p* < 0.001) and RCI fish (*p* < 0.03; Figure 14E)]. Both *igd* membrane bound and secretory forms were greater expressed in the RI fish at every time point in comparison to the other treatments (Figure 14F). In this treatment (RI), *igd mem* was significantly upregulated at 50 (*p* < 0.04) and 80 dpe (*p* = 0.02) relative to the control. The mRNA levels of *igd sec* in the RI fish were also significantly elevated in comparison to the control fish at 80 dpe (*p* < 0.01; Figure 14G).

## 4 DISCUSSION

Host populations are rarely homogenous, therefore, to truly understand the ultimate effect of a disease on a population, the impact on different host phenotypes must be determined. In the present study, we explored the hosts’ response to parasite encounters in phenotypes with (a) different ages and (b) with heterogeneous infection life histories. Our first prediction concerned the role of age on immunity and, the results confirmed our expectations. In younger fish (YOY infected), the parasite proliferated quicker, and disease severity was increased, thus PKD appeared more profound in these fish. This observation can be substantiated by the relative lower infection tolerance and the difference in immune response patterns, which may indicate reduced or immature immunocompetence in younger fish. Previous PKD research has focused almost exclusively on YOY fish due to supposed increased mortality and indeed, from our results it would appear there is an ontogenetic decline in *T. bryosalmonae* susceptibility.

Our second prediction concerned immunity in persistently infected (PI) and re‐infected fish (RI). This experiment also consisted of fish that either cleared or avoided infection completely (CI) and fish that were re‐exposed and tested negative for the parasite (RCI). From this perspective, it appears that the population may contain fish that either avoid infection completely, clear the infection and can avoid re‐infection or are persistently infected that are susceptible to re‐infection. In the infected fish (PI and RI) parasite burden, disease severity and infection tolerance were comparable. In fact, the PI fish and RI fish had lower relative tolerance compared to the juvenile 1+ fish infected for the first time. Hence, the long‐term (persistent) infection experienced by these fish resulted in overall lower host health, in contrast to fish of the same age infected over a shorter time (juvenile 1+ infected). Both PI and RI fish continuously released fish malacospores and simultaneously maintained an active adaptive immune response throughout the study. This suggests that constant parasite replication and development occurs in the host as determined by fish malacospore release and stable parasite intensity measurements, and that these processes may provide a perpetual source of parasite antigens that stimulates the host immune response and through this process might serve to maintain host immunity. Furthermore, several noteworthy variations were observed in the immune response patterns between the infected treatments, such as the stronger expression in the RI fish of all secretory Ig transcripts (*igm*, *igt*, *igd*) and in both secretory and membrane isoforms of *igd* relative to the PI, CI, RCI treatments. Research in mammals with persistent parasite infections tell us some hosts may maintain protective immunity to repeat infections via concomitant immunity, which results in amelioration of disease pathology without clearance of either the persistent or the incoming parasite (Mandell & Beverley, 2016, 2017; Sacks, 2014). Likewise, our findings would also point towards a form of concomitant immunity that occurred in the RI fish.

Host age can play a pivotal role in establishing both the risk and outcome of infection in numerous disease systems (Bundy et al., 1987; Duerr et al., 2003; Gregory, 1992; Mpofu et al., 2020; Phillips et al., 2020). Evidence exists that teleost fish can mount a humoral immune response very early in ontogeny (Magnadottir et al., 2005). However, a study of white bass and striped bass hybrids (*Morone chrysops* × *Morone saxatilis*) juveniles aged between 4 and 19 months found lower antibody levels in younger juvenile fish after vaccine challenge (Hrubec et al., 2004). Hence, despite presence of adaptive immunity in YOY infected fish, full intrinsic maturation and immunocompetence might not come till later in life. A reason for the reduced immunocompetence in younger fish might be due to greater physiological investment in other priorities such as growth and maintenance. For example, owing to the size of the YOY fish it could be expected that they might prioritize growth over an immune response in contrast to the larger juvenile fish, in which growth might be slowing. However, there were only subtle differences between the specific growth rates (SGR). In fact, the SGR was greater in both groups of infected fish at 80 dpe, a time that could be classed the clinical phase of PKD. This could be due to the pathological reaction during PKD as the posterior kidney weights were increased and this may occur in other impacted organs (anterior kidney, spleen and liver). This aside, infection did not strongly impact host growth in any of the experimental groups relative to uninfected fish. Our results reinforce a recent report in which no correlation was reported between the *T. bryosalmonae*‐induced immune response and a reduction of growth in juvenile rainbow trout (Wernicke von Siebenthal et al., 2018). Essentially, this highlights the dynamism of ectotherms and their capability in managing multiple energy demanding processes. Still, this could be pathogen‐dependant and may change during an acute infection.

During PKD pathogenesis rainbow trout exhibit a chronic lymphoid immunopathology mediated by a decrease in myeloid cells and a massive B cell dysregulation and proliferation with aberrant immunoglobulin and plasma cell production (Abos et al., 2018; Bailey, Segner, Casanova‐Nakayama, et al., 2017; Bailey, Segner, et al., 2020; Chilmonczyk et al., 2002). An imbalance of Th‐like responses and hyper‐production of *il‐10* has also been reported (Bailey, Holland, et al., 2020; Bailey, Segner, Casanova‐Nakayama, et al., 2017; Bailey, Segner, et al., 2020; Gorgoglione et al., 2013). In addition, strong expression of *igm* isoforms and *blimp1* has been reported in brown trout (Bailey, Strepparava, et al., 2019; Kumar et al., 2015; Sudhagar et al., 2019). Particularly heavy *il‐10* production has been noted in several fish‐myxozoan systems (Bjork et al., 2014; Korytář et al., 2019; Piazzon et al., 2018). In brown trout we also observed a strong B cell antibody type response in all infected fish. In the current study, the expression of *blimp1* and *il‐10* was greatly elevated in YOY infected fish in comparison to juvenile 1+ infected fish, with *blimp1* transcripts being greater elevated in YOY infected fish, than those with a persistent infection (PI) or those re‐infected (RI). Transcript profiles of the three fish Igs also appeared generally lower in the YOY infected fish relative to all other infection groups, which correlated with increased parasite loads. Taken together, these expression patterns might indicate a reduced immunocompetence of the adaptive immune response of younger fish in our study that likely resulted in greater pathogen burdens. Both *blimp1* and *il‐10* are key regulators in a vast amount of lymphocyte processes and their over expression could contribute to chronic lymphoid immunopathology of PKD. From this perspective, overproduction of *il‐10* is a key determinant of enhanced immunopathology in several parasite diseases of humans (Iyer & Cheng, 2012), and *blimp1*, has been shown to play an important role in *il‐10* production (Cretney et al., 2011).

In all infected fish, *igt* was the most dominant Ig isoform and this agrees with investigations of rainbow trout (Abos et al., 2018; Gorgoglione et al., 2013). In general, in the RI fish, there seemed to be a stronger upregulation of the secretory Igs in contrast to PI, CI and RCI fish. Meanwhile, *igd* transcripts were greater expressed in all older infected fish relative to YOY infected fish. The strongest expression of both secreted and membrane *igd* occurred in the RI fish. This suggests a putative protective role of *igd* during PKD pathogenesis and this is something that deserves further investigation. Especially considering that a previous gene screening of naive juvenile rainbow trout found both membrane and secretory forms of *igd* were refractory to PKD and that the immunoprotective role of *igd* in teleosts remains largely unknown (Gorgoglione et al., 2013). While *igm sec* was upregulated at 50 and 80 dpe in both YOY and juvenile 1+ infected fish and in RI fish at these time points. Surprisingly, *igm mem* did not show any elevated expression in YOY or juvenile 1+ infected fish, nor was it strongly upregulated in the other experimental groups. In rainbow trout naturally infected with *T. bryosalmonae*, *igm mem* was only lowly expressed or downregulated (Gorgoglione et al., 2013), however in an experimental lab exposure with the same species *igm mem* was strongly elevated in both the anterior and posterior kidney at 8 weeks post exposure (Bailey, Segner, et al., 2020). In this context, it is difficult to interpret our *igm mem* data particularly in the fish infected for the first time, as this result aside, collectively the B cell markers are strongly involved in the immune response.

Concerning the T cell markers assessed here, interestingly we found that *cd8β* was lowly expressed in juvenile 1+ infected fish but not in YOY infected fish at 50 and 80 dpe, whereas *cd8α* expression was upregulated similarly at these time points in both treatments. It is well known that different cell types can also express these *cd8* chains, providing different patterns of expression for the two genes. For example, at the inflammatory site of The authors suggested that these were reminiscent of mammalian cd8αα+ cells that are similar to dendritic cells. Therefore, one possible explanation could be that also in brown trout, some cells different than T cells express *cd8α*. However, as we had no measurements of dendritic cell markers in the present study, it is not possible to elaborate on this. Nevertheless, to characterize these differences (between the alpha and beta chain) is an interesting question for future research and one that requires more consideration.

Alternatively, variations in the response of the different host phenotypes should be envisioned from the parasite perspective. From this viewpoint, parasites are undoubtedly notorious immunomodulators (Harnett, 2014) and it could be hypothesized that *T. bryosalmonae* can disturb the functionality of B cell subsets and induce *il‐10* over production and that this is more pronounced in younger naive fish. In fact, parasite virulence has been demonstrated to evolve according to host phenotype in several studies (Gupta et al., 1994; Lagrue et al., 2007; Pfennig, 2001) and in the present study, all measurements of parasite infection success were increased in YOY fish; infection prevalence, parasite intensity and fish malacospore release. Accordingly, heterogeneity in host infection history status can pose different challenges to parasites and only a single level of virulence may not be appropriate for all host phenotypes (Pfennig, 2001). As a result, it could be advocated PKD is more severe in these YOY infected fish and that this might be linked to altered *T. bryosalmonae* virulence conferring to host phenotype.

A further explanation for the differences we observed in the YOY infected phenotype, might be due to the reduced size of the target organs, impacting parasite activity. The reduction in available space for parasite colonization could drive increased competition for limited host resources and place greater physiological demands on the host impacting all aspects of the infection. Intraspecific conflict between parasites has been well‐documented, resulting in competition through direct aggressive interactions or indirectly through immune cells, or via the consumption of shared host resources, such as space and nutrients (Wale et al., 2017). Moreover, intraspecific competition can also cause an increased rate of virulence and impact disease outcome (Alizon et al., 2013; Brown et al., 2009). On the contrary, in larger fish, the increased kidney capacity may help manage the parasite invasion via diluting the parasite concentration through the increased space and resources. While, in the wild, much larger fish might also confer a behavioural advantage/adaptation to avoiding parasites. For example, their size may enable them to stay in areas with higher water flow rates as has been reported for larger fish (Hockley et al., 2014). In this context, it has been suggested that often lower flow regimes are associated with the environmental parameters linked with an increased abundance of *T*. *bryosalmonae* malacospore release from the bryozoan host (Hutchins et al., 2021).

In the present study, fish negative for *T. bryosalmonae* had no parasite‐induced pathology (CI), and fish that were infected and when re‐exposed to the parasite, tested negative for *T. bryosalmonae* and had no parasite‐induced pathology (RCI). These fish may have been able to avoid infection completely or cleared the infection and had protective immunity to re‐exposure. In the present experiment, we were unable to determine which of these explanations is valid. In our earlier work, exploring protective immunity during PKD pathogenesis in rainbow trout it was found that in a re‐exposure scenario fish are either able to avoid re‐infection completely or mount an earlier more efficient immune response that minimized pathogen burden and resulted in a milder asymptomatic version of the disease (Bailey, Segner, & Wahli, 2017). In that study the authors did not investigate the immune response in those fish re‐exposed that were uninfected due lower number of sample availability. However, in the present study there were no strong indications of a distinct immune response pattern in either phenotypes (CI and RCI) and results were similar to juvenile 1+ control fish. Of course, it could be critiqued that (a) we did not measure the right immune genes. Or (b) that these fish for some reason did not encounter parasites during the exposure; (c) that the timing of the tissue sampling was too late to measure the protective immune response; or (d) that we sampled the wrong organ to measure the protective immune response. From the perspective of (d), we analysed the posterior kidney, yet putatively there could be a response in the gills, skin or blood which acts as a barrier preventing the parasite getting to the kidney. It has been well‐established that the gill is a major organ for antibody secreting cells and that the gill microbiota is predominantly coated with *igt* which is the most dominate Ig present during PKD pathogenesis (Xu et al., 2016). Thus, it may be that the parasite is neutralized before reaching the kidney. Within this framework, data from gastro helminths *Strongyloides* (Yasuda et al., 2014) and *N. brasiliensis* (Thawer et al., 2014) have shown that host protection to re‐infection do not always occur in the target organ, but at an earlier barrier. Both these parasites colonize areas of the intestine, but a protective immune response has been shown to occur in the lungs (Grencis, 2015). In any case, further experiments are necessary to expand upon this hypothesis as limited information exists on the gill response during any stage of PKD pathogenesis.

There were no major differences between RI and PI fish in terms of infection prevalence, parasite intensity, fish malacospore release, disease severity and infection tolerance. According to these parameters there is no net effect of the repeated parasite exposures, and that the population is only separated into phenotypes that clear infection or have a persistent infection. However, there were differences in the immune response patterns between PI and RI fish, particularly in terms of the intensity of the secretory Ig expression. It must be stated that all Ig isotypes were present in fish with a persistent infection, albeit at lower levels than in the RI fish. This implies that during a persistent infection and in a persistent infection post re‐exposure fish maintain long‐term humoral responses. In addition, both infection phenotypes were able to limit excessive tissue inflammation, as well as other health impacts that occur in severe clinical PKD. In the RI fish, this would align with the concept of concomitant immunity as an immune status in which prevention of re‐infection coincides with infection persistence from the original encounter. Still, evidence of concomitant immunity in fish is fragmentary at best, and clear descriptions of immunity in many systems can be paradoxical. Nevertheless, our findings highlight that a protective response on some level is achieved by the host, and this is something that had not been previously documented.

## 5 CONCLUSIONS

It is often the case that a comprehensive disease ecology framework for the understanding of pathogens that impact wild marine and freshwater fish is not readily available, despite a detailed database of fish immunology and pathology knowledge. In the present study, we find ourselves somewhere at the crossroads exploring heterogeneity in immune phenotypes. We highlighted several distinctive features of host immunity in this salmonid fish‐myxozoan‐parasite model not previously investigated. We demonstrated the relevance of heterogeneity in infection life history on disease outcomes. The immunity status of the differing phenotypes will also have net‐level population effects. This could be in terms of a dilution effect on the parasite acting almost in a herd immunity fashion, saturating infection biomass for the more susceptible individuals. This may be more pronounced in PKD, as theoretical studies of chronic infections have indicated that such individual variability in susceptibility in infection intensity may reduce the expected infection prevalence of parasites with lower transmissibility (White et al., 2018), given that there is no direct fish host–host transmission of *T. bryosalmonae*. Or alternatively an increase in older persistently infected fish, with greater dispersal capabilities relative to YOY fish might lead to an enhanced spreading of the disease. We identified several distinctive features of immune ontogeny and protective mechanisms in this salmonid fish‐myxozoan–parasite model not previously investigated. In either circumstance the relevance of such themes on a population‐level requires greater research in many aquatic disease systems to generate a comprehensive understanding and establish a universal framework for future studies. Undoubtedly, a clearer interpretation of within‐host processes will refine the subtleties of our understanding into how this disease impacts a population in terms of the spread and maintenance of the parasite.

## CONFLICT OF INTEREST

The authors declare no conflicts of interest.

## AUTHORS' CONTRIBUTIONS

C.B., T.W., N.S. and H.S. designed the experiments; C.B., C.T. and H.S. formulated the concept. N.S. collected the samples; C.B. and N.S. performed all experiments; C.B., C.T., H.P.‐S., N.S. and H.S. analysed the data; C.B., H.S. and C.T. wrote the main body of the manuscript; C.B. complied figures/tables. All authors edited, read and approved the final manuscript.

## Supporting information

Fig S1

Table S1

## Data Availability

All data are available within the article or supporting materials or available online via the Mendeley data depository: https://data.mendeley.com/datasets/rxfbkr6bsr/1 (Bailey, 2021).
