# Supplementary material for: It's a hard knock life for some: Heterogeneity in infection life history of salmonids influences parasite disease outcomes
Source: J Anim Ecol. 2021 Jul 21;90(11):2573–93. doi: 10.1111/1365-2656.13562 (PMC8597015; doi:10.1111/1365-2656.13562)
Supplement: Supplementary file 1 — Fig S1 [file JANE-90-2573-s002.docx]

**Figure S1**. Survival curves with S.E of (A) YOY infected fish and juvenile 1+ infected fish and (B) fish with a persistent infection and reinfected fish.
