# Supplementary material for: It's a hard knock life for some: Heterogeneity in infection life history of salmonids influences parasite disease outcomes
Source: J Anim Ecol. 2021 Jul 21;90(11):2573–93. doi: 10.1111/1365-2656.13562 (PMC8597015; doi:10.1111/1365-2656.13562)
Supplement: Supplementary file 2 — Table S1 [file JANE-90-2573-s001.docx]

| Gene | Acc. number | FWD | REV | Ref |
| --- | --- | --- | --- | --- |
| *elf1α* | XM_029735647.1 | GATCCAGAAGGAGGTCACCA | TTACGTTCGACCTTCCATCC | (1) |
| *il-1β* | XM_029716234.1 | AGTGCTGTGGAAGAACATATAGTGTTG | CATCAGGACCCAGCACTTG | (2) |
| *Il-10* | XM_014168417.1 | AGACTTCCCTGTTGGACGAA | CTGCGTTCTGTTGTTCATGGC | (2) |
| *cd4* | EU153044 | TGTGCTTGTGTATGGGCAGG | GGTGAAACGGAAGTTGGTT | (3) |
| *cd8a* | AY701523 | CCAAGTCGTGCAAAGTGGGAA | CTTGGCTGTCTTTTGTAATGATGTGG | (3) |
| *cd8b* | AY701524 | GAACTATCAAACCCCAGAAGGCTGTG | GACACTTTTTGGGTAGTCGGCTGAA | (3) |
| *blimp 1* | XM_029755613.1 | AGCTGTCCAACCTCAAGGTCC | TTGCGGCACACCTGGGCATTC | (4) |
| *igm mem* | AY748799.1 | CCTACAAGAGGGAGACCGATTGTC | GTCTTCATTTCACCTTGATGGCAGT | (5) |
| *igm sec* | AY748798 | TACAAGAGGGAGACCGGAGGAGT | CTTCCTGATTGAATCTGGCTAGTGGT | (3) |
| *igt mem* | GFIS01045963.1 | CTGTGGTCCACCACCTCCTC | \| TCTATCTCTGCAGTCCATCATGTGT \| \| --- \| | Here |
| *igt sec* | GFIS01045961.1 | AGTCCTTGGGTAACTCATGTGAAGA | AATTGGCTATTCTGACCCACAGA | Here |
| *igd mem* | AY748805.1 | TGAACATATCCAAACCAGAGCTCC | GTCCTGAAGTCATCATTTTGTCTTGA | (5) |
| *igd sec* | AY748802.1 | TGAACATATCCAAACCAGGTGTCTG | GTCCTGAAGTCATCATTTTGTCTTGA | (5) |

**Table. S1**. Sequences and corresponding accession numbers of brown trout primers used in this study.

**References**

1. Granja AG, Perdiguero P, Martín-Martín A, Díaz-Rosales P, Soleto I, Tafalla C. 2019 Rainbow trout IgM+ B cells preferentially respond to thymus-independent antigens but are activated by CD40L*. Frontiers Immunol*, **10**, 2902. (doi:10.3389/fimmu.2019.02902)

2. Bailey C., Strepparava N., Wahli T., Segner H. 2019 Exploring the immune response, tolerance and resistance in proliferative kidney disease of salmonids. *Dev Comp Immunol,* **90**, 165-175. (doi:10.1016/j.dci.2018.09.015)

3. Gorgoglione B., Tiehui W., Secombes C.J., Holland J.W. 2013 Immune gene expression profiling of Proliferative Kidney Disease in rainbow trout *Oncorhynchus mykiss* reveals a dominance of anti-inflammatory, antibody and T helper cell-like activities. *Vet Res,* **44**, 1144-1155. (doi: 10.1186/1297-9716-1144-1155)

4. Díaz-Rosales, P., Bird, S., Wang, T.H., Fujiki, K., Davidson, W.S., Zou, J. and Secombes, C.J., 2009. Rainbow trout interleukin-2: cloning, expression and bioactivity analysis. *Fish Shellfish Immunol*, ***27***, 414-422. (doi:10.1016/j.fsi.2009.06.008)

5. Attaya, A., Wang, T., Zou, J., Herath, T., Adams, A., Secombes, C.J. and Yoon, S., 2018. Gene expression analysis of isolated salmonid GALT leucocytes in response to PAMPs and recombinant cytokines. *Fish Shellfish Immunol*, ***80***, 426-436. ([doi:10.1016/j.fsi.2018.06.022](https://doi.org/10.1016/j.fsi.2018.06.022))
